# The Light vs. Dark Triad of Personality: Contrasting Two Very Different Profiles of Human Nature

**DOI:** 10.3389/fpsyg.2019.00467

**Published:** 2019-03-12

**Authors:** Scott Barry Kaufman, David Bryce Yaden, Elizabeth Hyde, Eli Tsukayama

**Affiliations:** ^1^Positive Psychology Center, University of Pennsylvania,, Philadelphia, PA, United States; ^2^Business Administration Division, University of Hawai‘i-West O‘ahu, Kapolei, HI, United States

**Keywords:** dark triad, antagonism, light triad, personality, positive psychology

## Abstract

While there is a growing literature on “dark traits” (i.e., socially aversive traits), there has been a lack of integration with the burgeoning research literature on positive traits and fulfilling and growth-oriented outcomes in life. To help move the field toward greater integration, we contrasted the nomological network of the Dark Triad (a well-studied cluster of socially aversive traits) with the nomological network of the Light Triad, measured by the 12-item Light Triad Scale (LTS). The LTS is a first draft measure of a loving and beneficent orientation toward others (“everyday saints”) that consists of three facets: *Kantianism* (treating people as ends unto themselves), *Humanism* (valuing the dignity and worth of each individual), and *Faith in Humanity* (believing in the fundamental goodness of humans). Across four demographically diverse samples (*N* = 1,518), the LTS demonstrated excellent reliability and validity, predicting life satisfaction and a wide range of growth-oriented and self-transcendent outcomes above and beyond existing measures of personality. In contrast, the Dark Triad was negatively associated with life satisfaction and growth-oriented outcomes, and showed stronger linkages to selfish, exploitative, aggressive, and socially aversive outcomes. This exploratory study of the contrasting nomological networks of the Light vs. Dark Triad provides several ways forward for more principled and data driven approaches to explore both the malevolent and beneficent sides of human nature.

## Introduction

“*I still believe, in spite of everything, that people are truly good at heart.*” – Anne (Frank, 1995) (1947/, p. 332).

“*What’s one less person on the face of the earth, anyway?*” – Ted Bundy (as quoted in Leyton, 2003).

We all have, within each of us, both a light and a dark side. We all vary, however, in the extent to which we consistently exhibit light vs. dark patterns of thoughts, feelings and behaviors in our daily lives. For over the past 15 years, there has been a flurry of empirical research on a number of “dark traits” that are associated with ethically, morally, and socially aversive beliefs and behaviors (Moshagen et al., 2018). There is an emerging consensus that the “dark core” (or so-called “heart of darkness”) of these dark traits consists of an antagonistic social strategy characterized by high levels of interpersonal manipulation and callous behavior (Jones and Figueredo, 2013; Marcus et al., 2018; Moshagen et al., 2018).

While there are some newcomers on the dark trait scene (e.g., sadism and spitefulness), the most studied and validated dark traits are indexed by the now infamous “Dark Triad” of personality: narcissism, Machiavellianism, and subclinical psychopathy (Paulhus and Williams, 2002; Jonason et al., 2012b; Furnham et al., 2013; Muris et al., 2017). Since the initial paper proposing a Dark Triad of personality (Paulhus and Williams, 2002), research on the topic has increased every year, with two thirds of the publications on the Dark Triad appearing in 2014 and 2015 alone (Muris et al., 2017). While each of the three members of the Dark Triad have unique features and correlates (e.g., Jones and Figueredo, 2013; Glenn and Sellbom, 2015; Muris et al., 2017), there is enough overlap among these socially aversive personalities that researchers have argued that they “should be studied in concert” (Paulhus, 2014, p. 421).

Considering the dark core of the Dark Triad, it’s no surprise that the field has focused on predicting a wide range of aversive psychosocial outcomes, including *aggression and violence* (Pailing et al., 2014; Dinic and Wertag, 2018; Knight et al., 2018; Paulhus et al., 2018); *low affective empathy* (Wai and Tiliopoulos, 2012; Jonason and Kroll, 2014; Pajevic et al., 2018), *strong motives for self-enhancement, achievement, power, money, hedonism, and short-term instrumental sex* (Jonason et al., 2008; Lee et al., 2013; Kajonius et al., 2015; Jonason and Ferrell, 2016; Balakrishna et al., 2017), *counterproductive and coercive behaviors in the workplace* (O’Boyle et al., 2012; Spain et al., 2013; Jonason et al., 2014, 2018; Spurk et al., 2015); *a heartless “love style” characterized by high levels of infidelity, active prowling, game playing, practical utility, avoidant attachment style*, and a *preference for “one-night-stands” and “friends-with-benefits”* (Jonason and Kavanagh, 2010; Jonason and Buss, 2012; Jonason et al., 2012a; Koladich and Atkinson, 2016; Unrau and Morry, 2017; Alavi et al., 2018); and *immature defense mechanisms* (Richardson and Boag, 2016). Furthermore, as if the preceding findings were not socially aversive enough, the Dark Triad has even been associated with committing the “seven deadly sins” more often (Veselka et al., 2014; Jonason et al., 2017).

While this growing research base has contributed substantially to our understanding of the darker side of human nature, many fulfilling and growth-oriented outcomes possible in life have largely gone unexplored in the Dark Triad literature. The latest science of well-being includes a wide range of topics, including *positive emotions* (Fredrickson, 2001), *life satisfaction* (Diener, 1984; Diener et al., 1999), *personal growth* (Ryff, 1989; Ryff and Keyes, 1995), *altruism* (Keltner, 2009; Ricard, 2016), *gratitude* (Emmons and Crumpler, 2000; Emmons and McCullough, 2004), *savoring* (Kurtz, 2018), *forgiveness* (Witvliet and Luna, 2018), *intellectual humility and a quiet ego* (Wayment et al., 2014; Zachry et al., 2018), *hope* (Snyder et al., 2003; Lopez, 2013), *courage* (Pury and Saylors, 2018), *mindfulness* (Kabat-Zinn, 2013; Langer and Ngnoumen, 2018), *positive connection* (Fredrickson, 2013), *positive romantic relationships* (Pawelski and Pawelski, 2018), *competence* (Ryan and Deci, 2000), *grit* (Duckworth et al., 2007), *self-efficacy* (Bandura, 1977), *healthy self-esteem* (Tafarodi and Swann, 1995; Kernis, 2003), *autonomy* (Ryan and Deci, 2000), *meaning* (Steger et al., 2006; Martela and Steger, 2016), *purpose* (Damon, 2009), *engagement and flow* (Csikszentmihalyi, 2008), *awe, self-transcendence, and spirituality* (Keltner and Haidt, 2003; Yaden et al., 2016, 2017a,b, 2018), *morality* (Meindl et al., 2015; Jayawickreme and Fleeson, 2017), *character strengths* (Peterson and Seligman, 2004), *mature coping styles* (Vaillant, 1998), and *authenticity* (Kernis and Goldman, 2006; Wood et al., 2008).

Therefore, while it is certainly true that there is a malevolent side of human nature, and the Dark Triad literature has contributed important information to our understanding of this aspect of humanity, research has also clearly articulated a positive, growth-oriented side of human beings – a beneficent side (Maslow, 1962; Rogers, 1961; Seligman, 2012). Too much focus on one aspect of human nature at the expense of the other misrepresents the full capacities of humanity (see Maslow, 1962). What is missing in the field, we believe, are empirical investigations that include measures of the dark side *and* measures of the light side, and that look at both maladaptive outcomes and well-being-related variables, all in the *same* study. The main aim of the current investigation is an attempt to do just that – to help further integration between two fields that been traveling mostly on separate paths.

To explore the contrasts between the dark and light side of personality, we created a first draft of a brief measure of the light side of personality that could provide a useful direct contrast to the common core of dark traits. In constructing the scale, we were motivated by the question: *what would an everyday loving and beneficent orientation toward others look like that is in direct contrast to the everyday antagonistic orientation of those scoring high on dark traits?* To inform the construction of items, we looked at existing measures of the Dark Triad (the most well-validated cluster of dark traits that have been studied) and generated a variety of items that conceptually represented the opposite interpersonal orientation toward others, ensuring that none of the items were merely reverse-coded versions of those comprising the Dark Triad scales.

In addition to increasing integration between literatures that rarely talk to each other, we believe the current investigation can also inform existing debates in the Dark Triad literature. It is well-known that the Dark Triad is negatively associated with Big Five Agreeableness (e.g., Jonason et al., 2013) and is even more strongly inversely correlated with the HEXACO Honesty-Humility factor of normal personality variation, which consists of the facets of sincerity, fairness, greed avoidance, and modesty (Ashton et al., 2000; Lee and Ashton, 2005; Jonason and McCain, 2012; Book et al., 2015; Hodson et al., 2018). The extent to which the Dark Triad out-predicts important life outcomes above and beyond these “normal” facets of personality remains an open and interesting question, however. Also, while we expected that the Light Triad would show strong positive associations with these standard measures of the socially desirable personality, we predicted that the Light Triad would not be completely redundant with such measures. Therefore, in the current study we assessed the predictive validity of the Light and Dark Triad above and beyond both the HEXACO Honesty-Humility dimension of personality, as well as measures of Big Five Agreeableness.

Another interesting question that has not received as much attention in the Dark Triad literature is whether normal personality variation can predict meaningful outcomes after partialing out the antagonistic traits comprising the Dark Triad. For instance, recent research suggests that the agentic aspect of narcissism offers a protective factor, and that grandiose narcissism is even related to *adaptive* outcomes once the antagonistic aspect of narcissism is partialed out (Kaufman et al., 2018). The present study will be in a position to not only assess whether the Dark Triad is completely redundant with normal personality variation (disagreeableness and the inverse of honesty-humility), but also whether the residual variance in the Dark Triad is associated with *adaptive* outcomes.

Another controversy in the field is the extent to which the Dark Triad uniformly predicts adverse and transgressive psychosocial outcomes across the board. Are there any benefits to having a preponderance of Dark Triad characteristics? Through broadening the nomological network of the Dark Triad to include a number of adaptive outcomes in life, we believe the current paper is in a better position to assess the potential costs and benefits of both the dark *and* light side of personality. In order to reveal the nomological network surrounding this construct, we do not rely solely on self-report scales but we also include measures of moral judgment and offer participants opportunities to either donate bonus reward money to charity or keep it for themselves while playing an adaptation of the “Dictator Game.”

### Current Studies

In this series of studies, we sought to (a) to expand the already-existing nomological network of the Dark Triad, and (b) to establish a nomological network for the Light Triad, by examining relationships with a wide variety of outcomes. Based on the prior literature, we expected the Dark Triad to generally be positively related to maladaptive, selfish, and aggressive outcomes, and negatively related to more prosocial, growth-oriented, and self-transcendent outcomes, and for the Light Triad to generally show the opposite pattern of results. We also believed that some relationships would diverge from this general pattern, as these two constructs are not merely opposites of each other. While primarily exploratory, we expected a that the light and dark triad would show the strongest discrimination when it comes to the core motives and values underlying each trait (e.g., self-enhancement motives and values vs. self-transcendent motives and values) and worldview (e.g., a fundamental belief in the goodness of humans and acceptance of others). We also expected that the light triad would show a positive relationship to the facets of interpersonal guilt that most strongly reflect an empathetic concern for others (O’Connor et al., 1997).

In sum, our main aims were to show (1) that the Light Triad can be measured with a reliable scale, (2) that the Light Triad is distinct from the inverse of the Dark Triad, Big Five Agreeableness, and the Honesty-Humility sub-scale of the HEXACO, (3) that the Light Triad predicts well-studied positive and negative outcomes over and above Agreeableness and Honesty-Humility, and (4) that the Light Triad is a useful explanatory construct informing both the literatures on well-being and the Dark Triad.

## Materials and Methods

### Participants

A total of 1,518 participants were recruited across four demographically diverse samples using two different data collection platforms. Sample size for each study was determined based on the minimal number of participants that would provide meaningful individual differences results (*N* > 150). All four studies received IRB approval from the University of Pennsylvania. Participants for the first two studies (described in this paper as “Study 1” and “Study 2”) were recruited from Amazon’s Mechanical Turk (M-Turk), with the restriction that all participants are currently living in the United States and were above the age of 18. Participants for the other studies (described in this paper as “Study 3” and “Study 4”) were recruited from a newer platform called Prolific Academic. Research shows that Prolific Academic is a viable alternative to Mechanical Turk (Peer et al., 2017). While the data quality of Prolific Academic has been found to be comparable to Mechanical Turk, participants recruited from Prolific Academic have been found to be more diverse and less dishonest compared to participants recruited from M-Turk (Peer et al., 2017). Even so, M-Turk samples have been found to be generally representative and adequate as well (Buhrmester et al., 2011). All studies took participants about 25 min on average to complete.

*Study 1* comprised 387 M-Turk participants with an average age of 34.60 (*Min* = 19, *Max* = 72, *SD* = 10.16). There was about an equal gender split (Male = 50.4%, Female = 49.6), and 80% identified as White (with the rest of the sample mostly identifying as either Hispanic, Latino, Black, or Asian). *Study 2* comprised 670 M-Turk participants with an average age of 36.07 (*Min* = 19, *Max* = 74, *SD* = 11.82). There was also about an equal gender split (Male = 47.5%, Female = 52.5%), and 80% identified as White (with the rest of the sample mostly identifying as either Hispanic, Latino, Black, or Asian). *Study 3* comprised 267 Prolific Academic participants, with an average age of 36.02 (*Min* = 18, *Maximum* = 77, *SD* = 12.42). The sample consisted of more females (58.1%) than males (41.9%), and 89% identified as White (with the rest of the sample mostly identifying as either Hispanic, Latino, Black, or Asian). *Study 4* consisted of 194 participants with an average age of 34.73 (*Min* = 18, *Max* = 66, *SD* = 11.13). There was an almost equal gender split (Male = 49%, Female = 51%), and 84% identified as White (with the rest of the sample mostly identifying as either Hispanic, Latino, Black, or Asian). While 187 participants recruited from Prolific Academic across Studies *3* and *4* came from the United States, 267 came from the United Kingdom and Ireland, thus suggesting that our results may generalize across at least some cultures.

### Measures

In each study, participants completed a battery of self-report survey items and tasks. For clarity of presentation, we grouped descriptions of the tests by conceptual category and present the results using the same grouping. Specific details about which test was included in which study, and the order of presentation of the tests, can be found in Table 1. Information about which studies each test came from is also indicated in the tables provided in the Section “Results.” For more information about each scale, including example items, please see the Supplementary Materials.

**Table 1:** Order of presentation of tests for each study.

Study 1 (*n* = 387)	Study 2 (*n* = 670)	Study 3 (*n* = 267)	Study 4 (*n* = 194)


Demographics	Demographics	Demographics	Demographics


The Dark Triad of Personality (D3- Short; Jones and Paulhus, 2014)	The Big Five Aspect Scales (BFAS; DeYoung et al., 2007)	The Big Five Inventory—2 (BFI-2; Soto and John, 2017)	The Big Five Inventory—2 (BFI-2; Soto and John, 2017)


Light Triad Scale (36 items)	The HEXACO Personality Inventory-Revised-Honesty Humility (HEXACO-60, Ashton and Lee, 2009)	Light Triad (36 items)	Light Triad (16 items)


Social Desirability (Strahan and Gerbasi, 1972)	The Cognitive, Affective, and Somatic Empathy Scales (CASES, Raine and Chen, 2018)	The Five-Factor Narcissism Inventory-Short Form (FFNI-SF)	The Dark Triad of Personality (D3- Short; Jones and Paulhus, 2014)


The HEXACO Personality Inventory-Revised-Honesty Humility (HEXACO-60, Ashton and Lee, 2009)	Light Triad Scale (16 items)	The Psychopathic Personality Inventory – Short Form (PPI-SF; Tonnaer et al., 2013)	Social Desirability (Strahan and Gerbasi, 1972)


The Dispositional Positive Emotion Scales Questionnaire-Compassion (DPES, Shiota et al., 2006)	Social Desirability (Strahan and Gerbasi, 1972)	The Triarchic Personality Measure (TriPM; Patrick, 2010)	The Authenticity Scale (TAS; Wood et al., 2008)


The Big Five Inventory—2 (BFI-2; Soto and John, 2017)	The Dark Triad of Personality (D3- Short; Jones and Paulhus, 2014)	The Interpersonal Guilt Questionnaire (ICQ; O’Connor et al., 1997)	The Authenticity Inventory (AI-3; Kernis and Goldman, 2006)


The Revised Sociosexual Orientation Inventory (SOI-R, Penke and Asendorpf, 2008)	Reactive-Proactive Aggression Questionnaire (RPQ, Raine et al., 2006)	The Defense Style Questionnaire (DSQ; Andrews et al., 1993)	The Rosenberg Self-Esteem Scale (RSES; Rosenberg, 1965)


The Love Attitudes Scale (LAS, Hendrick and Hendrick, 1986)	Utilitarian Moral Dilemmas (items adapted from Glenn et al., 2009)	The Adult Attachment Scale– Revised (AAS; Collins, 1996)	The Contingencies of Self Worth Scale (CSW, Crocker et al., 2003)


The Unified Motives Scales (UMS, Schönbrodt and Gerstenberg, 2012)	The Selfishness Questionnaire (SQ; Raine and Uhi, 2018)	Social Desirability (Strahan and Gerbasi, 1972)	The Adult Attachment Scale– Revised (AAS; Collins, 1996


The Balanced Measure of Psychological Needs Scale (BMPN, Sheldon and Hilpert, 2012)	Dictator Game (adapted from Eckel and Grossman, 1996)	The Cognitive Triad Inventory (CTI; Beckham et al., 1986)	The Sense of Self Scale (SOSS; Flury and Ickes, 2007)


Unpredictability of Childhood (adapted from Mittal et al., 2015; Young et al., 2018)	Spiritual Experience (Yaden and Newberg, unpublished).	The Portrait Values Questionnaire-Revised (PVQ-RR, Schwartz et al., 2012)	The Values in Action (VIA) Brief Strengths Test (Peterson and Seligman, 2004)


The Satisfaction with Life Scale (SWLS, Diener et al., 1985)	The Varieties Scale (Yaden and Newberg, unpublished)	Beliefs about self and others (items created by authors)	The Curiosity and Exploration Inventory-II (CEI-II, Kashdan et al., 2009)


The Quiet Ego Scale (QES; Wayment et al., 2014)			The Epistemic Curiosity Scale (ECS; Litman and Spielberger, 2003)


The Conspicuous Consumption—Extra Money Scale (Lee et al. (2013))			The Death Transcendence Scale (DTS; Hood and Morris, 1983)


			Beliefs about self and others (items created by authors

#### Demographics

##### Annual income

Participants were asked to report how much they earned during the past 12 months.

##### Childhood income

Participants were asked to report their family’s financial situation when they were a child on a seven-point scale, ranging from “very poor” to “very rich.”

##### Education

Participants were asked to report their education level on a nine-point scale.

##### Unpredictability of childhood

We assessed this using eight items, three of which had been developed for use in a prior research study (Mittal et al., 2015), and five more were created for inclusion in a different study to better and more reliably measure the underlying construct on an unpredictable childhood environment (Young et al., 2018). Example items include, “My parents frequently had arguments or fights with each other or other people in my childhood.”

##### Social desirability

Strahan and Gerbasi (1972) validated a 13-item measure to assess and control for response bias in self-report research, which was employed in our studies.

#### Dark Triad Measures

##### The Dark Triad of Personality (D3- Short; Jones and Paulhus, 2014)

The Dark Triad of Personality (D3- Short; Jones and Paulhus, 2014) is a 27-item self-report questionnaire that measures Dark Triad traits, divided into three nine-item subscales: Machiavellianism (i.e., “It’s not wise to tell your secrets”), Narcissism (i.e., “People see me as a natural leader”), and Psychopathy, (i.e., “I like to get revenge on authorities”).

##### The Psychopathic Personality Inventory – Short Form (PPI-SF; Tonnaer et al., 2013)

The Psychopathic Personality Inventory – Short Form (PPI-SF; Tonnaer et al., 2013) is a 56-item version of the original 187-item PPI (Lilienfeld and Hess, 2001). Its eight-item Machiavellianism-Egocentricity facet was administered in this study in order to measure the Dark Triad trait of Machiavellianism.

##### The Triarchic Personality Measure (TriPM; Patrick, 2010)

The Triarchic Personality Measure (TriPM; Patrick, 2010) is used to assess the Dark Triad trait of Psychopathy.

##### The Five-Factor Narcissism Inventory-Short Form (FFNI-SF)

The Five-Factor Narcissism Inventory-Short Form (FFNI-SF) is the 60-item short form of the original Five-Factor Narcissism Inventory (FFNI; Glover et al., 2012), designed to assess the basic elements of narcissism from the perspective of a five-factor model.

#### Personality

##### The HEXACO Personality Inventory-Revised-Honesty Humility (HEXACO-60, Ashton and Lee, 2009)

The HEXACO Personality Inventory-Revised-Honesty Humility (HEXACO-60, Ashton and Lee, 2009) is one of six subscales comprising the 60-item HEXACO personality inventory. It contains 10 items, which are divided into four facets: *sincerity* (e.g., “I wouldn’t pretend to like someone just to get that person to do favors for me”), *fairness* (e.g., “I would never accept a bribe, even if it were very large”), *greed-avoidance* (e.g., “Having a lot of money is not especially important to me”), and *modesty* (e.g., “I want people to know that I am an important person of high status”).

##### The Big Five Inventory—2 (BFI-2; Soto and John, 2017)

The Big Five Inventory—2 (BFI-2; Soto and John, 2017) is a 60-item scale that measures three more-specific facets for each of the Big Five domains of personality: Extraversion, Agreeableness, Conscientiousness, Negative Emotionality, and Open-Mindedness.

##### The Big Five Aspect Scales (BFAS; DeYoung et al., 2007)

The Big Five Aspect Scales (BFAS; DeYoung et al., 2007) is a 100-item scale that measures two aspects of each Big Five factor.

#### Psychological Needs and Motives

##### The Balanced Measure of Psychological Needs Scale (BMPN, Sheldon and Hilpert, 2012)

The Balanced Measure of Psychological Needs Scale (BMPN, Sheldon and Hilpert, 2012) is an 18-item questionnaire, containing three six-item subscales to evaluate the degree of satisfaction and dissatisfaction of three basic psychological needs: *autonomy*, *competence, and relatedness*.

##### The Unified Motives Scales (UMS, Schönbrodt and Gerstenberg, 2012)

The Unified Motives Scales (UMS, Schönbrodt and Gerstenberg, 2012) is a 40-item measure of four explicit motives: achievement, power, affiliation, and intimacy.

#### Values and Character Strengths

##### The Portrait Values Questionnaire-Revised (PVQ-RR, Schwartz et al., 2012)

The Portrait Values Questionnaire-Revised (PVQ-RR, Schwartz et al., 2012) consists of 57 items, designed to measure the 19 values that are differentiated in Schwartz et al.’s refined theory of basic values (2012; enumerated below), which include a “self-transcendence” factor, a “self-enhancement” factor, an “openness to change” factor, and a “conservation” factor. The need to save face and humility, which didn’t fit cleanly into any of the other categories, were analyzed separately.

##### The Values in Action (VIA) Brief Strengths Test (Peterson and Seligman, 2004)

The Values in Action (VIA) Brief Strengths Test (Peterson and Seligman, 2004) is a 24–item self–report questionnaire that measures the degree to which respondents endorse personal character strengths and virtues. There is a total of 24 strengths of character in the VIA Classification, upon which this scale is based.

#### Defense Styles

##### The Defense Style Questionnaire (DSQ; Andrews et al., 1993)

The Defense Style Questionnaire (DSQ; Andrews et al., 1993) is a 40-item questionnaire that measures “defense styles” based on the DSM-III-R draft glossary of defense mechanisms (Advisory Committee on Defense Mechanisms (Work Group to Revise DSM-III), 1986). This study used a revised version of the original 72-item DSQ (Andrews et al., 1989).

#### Worldview

##### The Cognitive Triad Inventory (CTI; Beckham et al., 1986)

The Cognitive Triad Inventory (CTI; Beckham et al., 1986) is a 30-item questionnaire that measures the “cognitive triad” of negative beliefs about one’s self, one’s future, and the world, which Aaron Beck argues is an important predictor of depression (e.g., Beck et al., 1979).

##### Beliefs

We asked participants to rate their agreement with the statements “Humans are good” and “I am good” on a five-point scale.

#### Self-Esteem and Authenticity

##### The Rosenberg Self-Esteem Scale (RSES; Rosenberg, 1965)

The Rosenberg Self-Esteem Scale (RSES; Rosenberg, 1965) is a 10-item index of global self-esteem that measures both positive and negative feelings about the self.

##### The Contingencies of Self Worth Scale (CSW, Crocker et al., 2003)

The Contingencies of Self Worth Scale (CSW, Crocker et al., 2003) is a 35-item scale assessing seven contingencies shown to be important internal and external sources of self-esteem. The subscales include *family support*, *competition*, *appearance*, *God’s love*, *academic competence*, *virtue*, and *approval from others*.

##### The Sense of Self Scale (SOSS; Flury and Ickes, 2007)

The Sense of Self Scale (SOSS; Flury and Ickes, 2007) is a 12-item measure that assesses the extent to which one has a weak versus strong sense of self. While the scale is unidimensional, it has items relating to four components of a weak sense of self: (1) *Tendency to confuse one’s feelings, thoughts, and perspectives with those of others*; (2) *Lack of understanding of oneself*; (3) *Sudden shifts in feelings, opinions, and values*; and (4) *Feeling of a tenuous existence*.

##### The Authenticity Scale (TAS; Wood et al., 2008)

The Authenticity Scale (TAS; Wood et al., 2008) is a 12-item scale that has three subscales: *authentic living*, *alienation from the self*, and *accepting external influence*.

##### The Authenticity Inventory (AI-3; Kernis and Goldman, 2006)

The Authenticity Inventory (AI-3; Kernis and Goldman, 2006) is a 45-item measure “conceptually designed to assess the unimpeded operation of one’s true—or core—self in one’s daily enterprise.” It contains four subscales: awareness, unbiased processing (reverse scored), behavior, and relational orientation.

#### Sex, Love, and Relationships

##### The Revised Sociosexual Orientation Inventory (SOI-R, Penke and Asendorpf, 2008)

The Revised Sociosexual Orientation Inventory (SOI-R, Penke and Asendorpf, 2008) is a nine-item self-report questionnaire designed to measure individual differences in the tendency to engage in sexual relationships without deeper emotional commitment. This scale is divided into three facets measured by the inventory: *behavior*—in terms of number of casual and changing sex partners, *attitude*—toward uncommitted sex, and *desire*—for people outside of one’s romantic relationship.

##### The Love Attitudes Scale (LAS, Hendrick and Hendrick, 1986)

The Love Attitudes Scale (LAS, Hendrick and Hendrick, 1986) is a 42-item questionnaire designed to measure attitudes toward love. The questionnaire combines attitudes toward one’s current (or else, recent or hypothetical) partner with attitudes about love in general. The scale is broken into six subscales (seven items each) that represent a different love style: *Eros*—passionate love, *Ludus*—game-playing love, *Storge*—friendship love, *Pragma*—practical love, *Mania*—possessive, dependent love, and *Agape*—altruistic love.

##### The Adult Attachment Scale– Revised (AAS; Collins, 1996)

The Adult Attachment Scale– Revised (AAS; Collins, 1996) is an 18-item scale that measures the attachment styles of adults. Consistent with Fraley and Spieker (2003), we computed two attachment styles: *anxious* (the extent to which a person is worried about being rejected or unloved) and *avoidant* (the extent to which a person avoids intimacy and feels he/she can depend on others to be available when needed).

#### Empathy, Compassion, and Interpersonal Style

##### The Dispositional Positive Emotion Scales Questionnaire-Compassion (DPES, Shiota et al., 2006)

The Dispositional Positive Emotion Scales Questionnaire-Compassion (DPES, Shiota et al., 2006) is one of seven subscales contained in the 38-item DPES self-report instrument. The five-item compassion subscale measures one’s dispositional tendency to feel compassion toward people in general.

##### The Cognitive, Affective, and Somatic Empathy Scales (CASES, Raine and Chen, 2018)

The Cognitive, Affective, and Somatic Empathy Scales (CASES, Raine and Chen, 2018) is a 30-item measure containing three subscales. We administered the 10-item cognitive empathy subscale, which refers to the capacity to cognitively understand how others feel and the 10-item affective empathy subscale, which refers to the capacity to experience the emotions of how others feel.

##### The Interpersonal Guilt Questionnaire (ICQ; O’Connor et al., 1997)

The Interpersonal Guilt Questionnaire (ICQ; O’Connor et al., 1997) is a 67-item scale that assesses four types of guilt: *survivor*, *separation*, *omnipotent responsibility*, and *self-hate*.

##### The Quiet Ego Scale (QES; Wayment et al., 2014)

The Quiet Ego Scale (QES; Wayment et al., 2014) measures “a self-identity that transcends egoism and identifies with a less defensive, balanced stance toward the self and others.” This 14-item scale is comprised of four subscales dedicated to measuring the following well- known psychological characteristics: detached awareness, inclusive identity, perspective taking, and growth.

#### Selfishness, Aggression, and Moral Judgment

##### The Conspicuous Consumption—Extra Money Scale (Lee et al., 2013)

The Conspicuous Consumption—Extra Money Scale (Lee et al., 2013) was constructed for use in a prior research experiment examining the relationships of sex, power, and money to Dark Triad characteristics. Participants were asked to indicate how they would spend an extra $100,000 on 13 items that represent either conspicuous consumption (e.g., luxury cars, high-end restaurant meals, etc.) or non-conspicuous consumption (e.g., health products, insurance, etc.). We obtained the final scale scores for this scale by subtracting non-conspicuous scale scores from conspicuous scale scores.

##### Reactive-Proactive Aggression Questionnaire (RPQ, Raine et al., 2006)

Reactive-Proactive Aggression Questionnaire (RPQ, Raine et al., 2006) is a 23-item, scale that measures the two-factor model of aggression, with 11 questions examining reactive aggression and 12 questions assessing proactive aggression.

##### Utilitarian Moral Dilemmas

Utilitarian moral decision-making was assessed using three condensed versions of high-conflict personal dilemmas— “Crying Baby,” “Footbridge,” and “Sacrifice” (Glenn et al., 2009). Participants were asked to rate how morally appropriate or inappropriate they found utilitarian actions (ones that are harmful but benefit the greater good).

##### The Selfishness Questionnaire (SQ; Raine and Uhi, 2018)

The Selfishness Questionnaire (SQ; Raine and Uhi, 2018) is a 24-item self-report instrument designed to measure selfish behaviors and attitudes. It is comprised of three subscales: egocentric, adaptive, and pathological.

##### Dictator Game

This is an experimental economic task in which participants decide how much, if any, of the money awarded to them by the experimenter they wish to give away to another recipient, without any negative consequences. Similar to Eckel and Grossman (1996) version of the dictator game, the described recipient was a charity foundation (*Save the Children*). Participants were informed that they would be given an additional $0.70 for their participation in the study and were asked how much they would be willing to donate to Save the Children.

#### Religion, Spirituality, and Self-Transcendence

##### Religious views

Participants were asked on a seven-point scale to report their degree of religiosity. These items were included as part of the demographics section in the beginning of the study.

##### Spiritual experience (Yaden and Newberg, unpublished)

We asked participants the following: “Have you had what you consider to be a spiritual experience? Spiritual experiences are generally considered brief, intense, and vivid subjective experiences involving perceiving an unseen order or connecting to something larger than yourself. People of all belief systems (e.g., secular, spiritual, and religious) report having had such experiences.”

##### The Varieties Scale (Yaden and Newberg, unpublished)

This scale is an operationalization of distinctions within William James’s *The Varieties of Religious Experience*. Participants were asked to indicate the extent to which they have had an experience involving a sense of unity (mystical factor) or of God/divinity (numinous factor).

##### The Death Transcendence Scale (DTS; Hood and Morris, 1983)

The Death Transcendence Scale (DTS; Hood and Morris, 1983) contains 23 items, based on the premise that “death is transcended through identification with phenomena more enduring than oneself.” Items are divided amongst five subscales: *mysticism*, *religious*, *nature*, *creative*, and *biosocial*.

#### Curiosity

##### The Curiosity and Exploration Inventory-II (CEI-II, Kashdan et al., 2009)

The Curiosity and Exploration Inventory-II (CEI-II, Kashdan et al., 2009) is a 10-item self-report instrument assessing individual differences in the recognition, pursuit, and integration of novel and challenging experiences and information. It contains two factors, with five items devoted to each: Stretching (about challenging oneself) and Embracing (accepting uncertainty).

##### The Epistemic Curiosity Scale (ECS; Litman and Spielberger, 2003)

The Epistemic Curiosity Scale (ECS; Litman and Spielberger, 2003) is a 10-item instrument developed specifically to assess individual differences in epistemic curiosity— the desire for new knowledge. The scale contains items to assess “interest” (I-type) curiosity, which is meant to “stimulate pleasurable feelings of situational interest.” It also contains items to assess “deprivation” (D-type) curiosity, which is meant to “relieve negative affective conditions of feeling deprived of knowledge.”

#### Life Satisfaction

##### The Satisfaction with Life Scale (SWLS, Diener et al., 1985)

The Satisfaction with Life Scale (SWLS, Diener et al., 1985) is a self-report instrument of five items to assess global life satisfaction.

## Results

### Light Triad Scale (LTS) Development

To construct the Light Triad Scale (LTS), we were informed by the themes of existing dark triad questionnaires (see “Materials and Methods” section) to create our own items. Our initial pool of 36 items (see Table 2) included a balanced mix of items that were conceptually the opposite of grandiose narcissism, Machiavellianism, and subclinical psychopathy, but which were not simply the reverse coded wording of already existing dark triad items. After constructing our initial pool of items, we consulted experts in positive psychology and personality psychology for their acceptance of the items as a reasonable contrast to the Dark Triad. We then conducted exploratory and confirmatory factor analysis to derive our final 12-item scale.

**Table 2:** Exploratory factor analysis loadings from the initial 36 light triad items in study 1.

	Factor			
Item	1	2	3	4
I don’t like hurting people’s feelings, even if it helps me achieve my goals.	**0.60**	0.13	0.20	-0.07
I don’t feel comfortable overtly manipulating people to do something I want.	**0.63**	-0.07	0.15	-0.04
I don’t expect people to treat me with more respect than others.	**0.62**	0.00	0.04	-0.02
I prefer honesty over charm.	**0.59**	0.09	-0.01	0.16
I don’t care much about being the center of attention.	**0.60**	-0.13	-0.09	0.15
If I found out I hurt someone’s feelings, I would feel guilty.	**0.51**	0.29	0.12	-0.15
I would like to be authentic even if it may damage my reputation.	**0.50**	0.11	0.08	0.09
I am not too bothered about my social standing.	**0.48**	0.02	-0.01	0.26
When I talk to people, I am rarely thinking about what I want from them.	**0.48**	0.05	0.27	0.14
I tend to be a careful person.	**0.36**	0.20	-0.13	0.18
I tend to be concerned with the dictates of my conscience.	**0.35**	**0.38**	-0.08	-0.12
Some people are more valuable than others.	-**0.35**	0.03	-0.27	0.11
If I see someone doing something good, I assume good intentions.	0.28	**0.34**	**0.31**	0.02
I rarely try to influence a specific outcome when I am interacting with someone.	**0.31**	-0.10	0.26	0.03
I’m not always on the look out for opportunities to advance my goals.	0.17	-0.09	0.09	-0.06
People feel comfortable around me.	-0.16	**0.62**	0.11	0.26
I enjoy listening to people from all walks of life.	0.08	**0.57**	0.14	0.08
I tend to applaud the successes of other people.	0.08	**0.53**	0.27	0.02
I tend to treat others as valuable.	0.16	**0.57**	0.12	-0.11
I easily make a connection with others.	-**0.30**	**0.48**	0.27	0.24
It is important to make sacrifices for my friends and family.	0.19	**0.51**	0.08	-0.05
I tend to be empathetic to other people.	0.26	**0.49**	0.18	-0.10
I tend to admire others.	-0.14	**0.47**	**0.36**	-0.14
I have dealt fairly with people I stand to benefit from.	**0.38**	**0.53**	-**0.31**	0.09
I tend to respect others I am competing against.	0.23	**0.45**	0.18	0.07
I’m the kind of leader who tends to bring out the best in others.	-0.22	**0.36**	0.22	0.25
I tend to see the best in people.	0.05	0.14	**0.71**	0.05
I tend to trust that other people will deal fairly with me.	0.06	0.05	**0.69**	0.01
I think people are mostly good.	0.04	0.12	**0.61**	0.03
I’m quick to forgive people who have hurt me.	0.12	-0.07	**0.65**	0.04
I am not afraid of sharing my weaknesses.	0.13	0.05	**0.41**	0.15
I tend to seek opportunities to contribute to society as a whole.	0.06	**0.33**	**0.36**	0.01
I have lost an opportunity due to my honesty.	0.21	0.19	-0.27	-0.09
My self-esteem is not dependent on what others think of me.	0.15	-0.10	0.05	**0.78**
I tend to value myself regardless of how others treat me.	-0.03	0.11	-0.03	**0.77**
I tend to feel guilty about my past moral failings.	0.23	0.17	-0.16	-**0.34**

*Loadings greater than 0.30 are bolded. Study 1 n = 387*.

#### Exploratory Factor Analyses (EFAs)

Because we expected the individual members of the Light Triad to be correlated, we used an oblique oblimin rotation with the fa function in the psych package in *R* on our Study 1 data (*N* = 387). To determine the number of factors to extract, we used parallel analyses (Horn, 1965), scree tests (Cattel, 1966), the minimum average partial criterion (Velicer, 1976), Bartlett’s chi-square test (Geweke and Singleton, 1980), and the Kaiser criterion (Kaiser, 1960). Except for the Kaiser criterion (which suggested seven factors), each of these tests suggested extracting four factors. The fourth factor (which reflected items measuring a secure self-esteem) only included 2 items with factor loadings above 0.4, so was therefore dropped as an error factor.

We settled on a three-factor solution. Based on the item content, we labeled the three factors: *Kantianism* (treating people as ends unto themselves, not as mere means to an end), *Humanism* (valuing the dignity and worth of each individual), and *Faith in Humanity* (believing in the fundamental goodness of humans). The label “Kantianism” was based on Immanel Kant’s second formulation of his categorical imperative: “Act in such a way that you treat humanity, whether in your own person or in the person of any other, never merely as a means to an end, but always at the same time as an end (Kant, 1785/1993, p. 36).” We thought Kantianism provided a sensible (and somewhat tongue-in-cheek) contrast to “Machiavellianism” within the Dark Triad framework.

To produce the most parsimonious scale possible, we selected four items with high factor loadings, adequate inter-item correlations, and face validity from each of the factors. We then conducted an iterative EFA on this reduced set of 12 items. The three-factor 12-item solution replicated the first three factors found in the previous solution (see Table 3). The observed internal consistency coefficients for the three 4-item factors were α = 0.82, α = 0.79, and α = 0.72, respectively. The observed internal consistency coefficient for the total scale was α = 0.84.

**Table 3:** Exploratory factor analysis loadings from select 12 light triad items in study 1.

	Factor		
Item	1	2	3
I tend to see the best in people.	**0.79**	0.06	0.00
I tend to trust that other people will deal fairly with me.	**0.80**	-0.04	-0.02
I think people are mostly good.	**0.75**	-0.02	0.02
I’m quick to forgive people who have hurt me.	**0.53**	-0.05	0.09
I tend to admire others.	0.08	**0.74**	-0.20
I tend to applaud the successes of other people.	-0.02	**0.74**	0.12
I tend to treat others as valuable.	-0.01	**0.60**	0.16
I enjoy listening to people from all walks of life.	0.06	**0.55**	0.10
I prefer honesty over charm.	-0.01	-0.02	**0.76**
I don’t feel comfortable overtly manipulating people to do something I want.	0.04	0.00	**0.57**
I would like to be authentic even if it may damage my reputation.	0.01	0.12	**0.57**
When I talk to people, I am rarely thinking about what I want from them.	0.23	0.09	**0.47**

*Loadings greater than 0.30 are bolded. Study 1 n = 387*.

#### Confirmatory Factor Analyses (CFAs)

**Figure 1:**
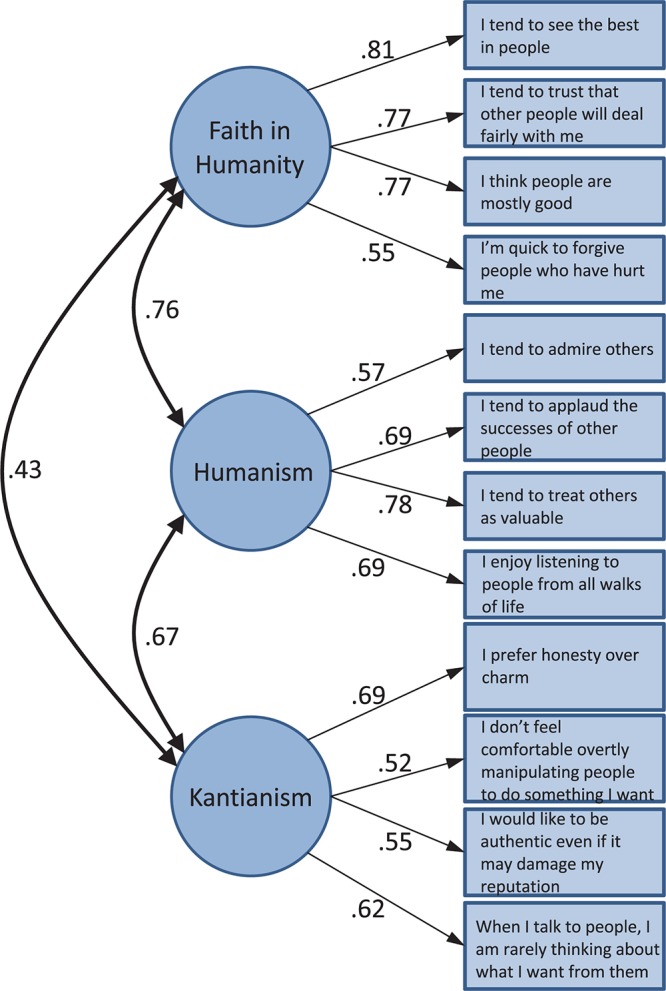
Confirmatory factor analysis on select 12 light triad items in Study 2. *n* = 670.

We next conducted confirmatory factor analyses (CFAs) on the Study 2 data (*n* = 670) to verify the solution identified by the iterative EFA on the Study 1 data. We estimated both a three-factor and a one-factor model, and found that the three-factor model fit the data significantly better than the one-factor model: Δχ^2^(3) = 440.44, *p* < 0.01. In the three-factor model, items were allowed to load freely on their respective factor, the factor loadings with other factors were set to zero, and the covariance between the factors was freely estimated. In the one-factor models, all items were allowed to load freely on a single factor. Factors were scaled by setting the variance equal to 1.0. All factor loadings were significant at *p* < 0.01. The three-factor model (see Figure 1) fit the data well—χ^2^(51) = 193.38, *p* < 0.01; CFI = 0.95; RMSEA = 0.065, 90% CI = [0.055–0.074]; SRMR = 0.05—whereas the one-factor model exhibited a poor fit to the data: χ^2^(54) = 633.82, *p* < 0.01; CFI = 0.78; RMSEA = 0.127, 90% CI = [0.118–0.136]; SRMR = 0.089.

**Figure 2:**
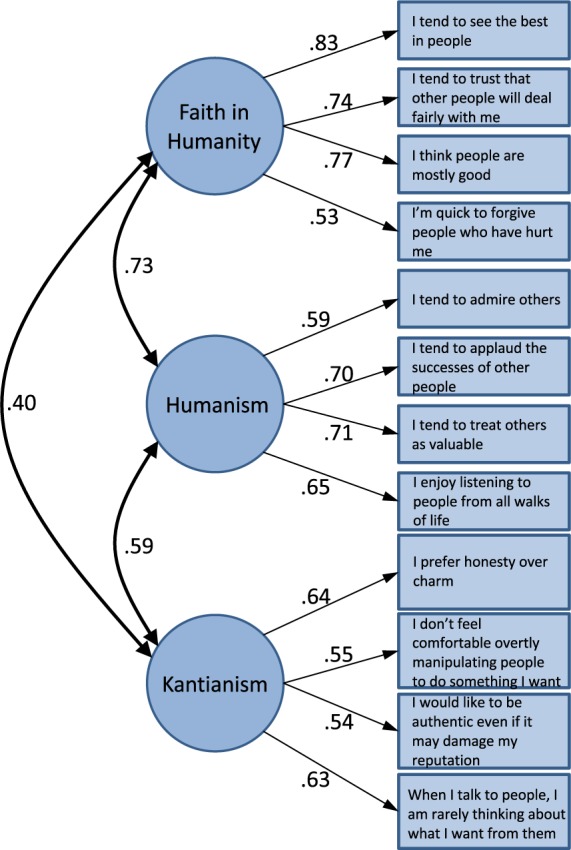
Confirmatory factor analysis on select 12 light triad items in combined sample. *N* = 1,518.

Finally, we conducted the same CFAs on the combined sample (*N* = 1,518) and replicated the CFA results (see Figure 2).

**Figure 3:**
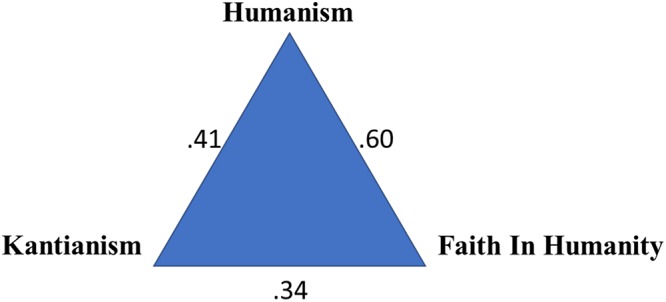
Correlations among light triad subscales. All correlations significant at *p* < 0.01. *N* = 1,518.

Specifically, the three-factor model fit the data significantly better than the one-factor model: Δχ^2^(3) = 889.61, *p* < 0.01. The three-factor model (see Figure 2) fit the data well—χ^2^(51) = 277.08, *p* < 0.01; CFI = 0.96; RMSEA = 0.054, 90% CI = [0.048–0.060]; SRMR = 0.04—whereas the one-factor model exhibited a poor fit to the data: χ^2^(54) = 1166.69, *p* < 0.01; CFI = 0.80; RMSEA = 0.117, 90% CI = [0.111–0.122]; SRMR = 0.083. Figure 3 shows the correlation among the three members of the Light Triad for all participants across all samples. The strongest correlation is between Humanism and Faith in Humanity (*r* = 0.60), and the weakest is between Kantianism and Faith in Humanity (*r* = 0.34).

The observed internal consistency coefficients for the three 4-item factors across all participants across samples (*N* = 1,518) were α = 0.80, α = 0.76, and α = 0.67, respectively. The observed internal consistency coefficient for the total scale across all participants was α = 0.84.

#### Relative Proportion of Light vs. Dark Triad Characteristics

**Figure 4:**
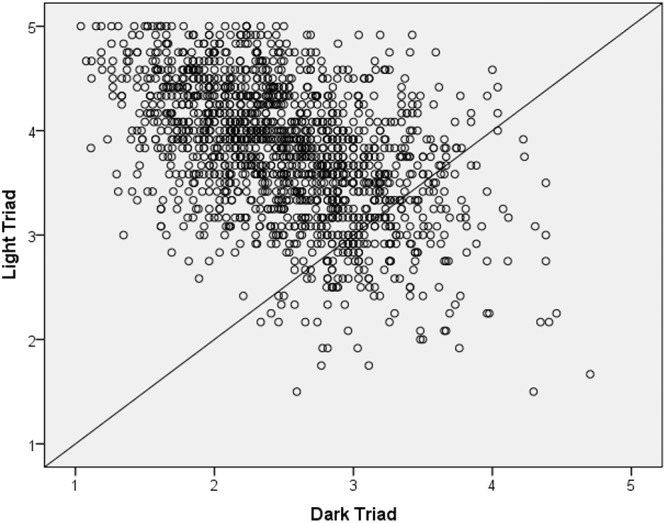
Scatterplot of light vs. dark triad scores in the total sample. *N* = 1518.

Figure 4 shows the scatterplot of Light vs. Dark Triad scores in the total sample (*N* = 1,518). The average Light Triad score for all participants across samples was 3.8 (Minimum = 1.5, Maximum = 5.00, *SD* = 0.64), whereas the average Dark Triad score for all participants across samples was 2.5 (Minimum = 1, Maximum = 4.7, *SD* = 0.62). A look at the scatterplot in Figure 4 suggests that extreme malevolence is rather rare in the general population.

We also calculated a LT vs. DT balance score for each participant by subtracting each person’s score on the Dark Triad from their score on the Light Triad. The average balance score for the entire sample was 1.3 (Minimum = -3.0, Max = 4., *SD* = 1.1), suggesting that the average person is tipped more toward the Light Triad relative to the Dark Triad.

#### Correlations Among the Light and Dark Triad

Overall, the Light Triad was moderately negatively correlated with the Dark Triad (*r* = -0.48, *p* < 0.01), suggesting that the Light Triad is not merely the opposite of the Dark Triad. While the rest of the analyses in this paper will focus on the total Light and Dark Triad scores, we thought it would be elucidating to look at the correlations among each of the factors of the Light and Dark Triad (see Table 4). For further regressions on each of these outcomes, please see the Supplementary Materials.

**Table 4:** Light triad total and subscale correlations with dark triad total and subscales.

	*M*	*SD*	1	2	3	4	5	6	7
(1) LT Total	3.80	0.64	-						
(2) LT Kantianism	4.01	0.75	0.71**	-					
(3) LT Humanism	3.94	0.74	0.83**	0.41**	-				
(4) LT Faith in Humanity	3.45	0.93	0.84**	0.34**	0.60**	-			
(5) DT Total	2.52	0.62	-0.48**	-0.56**	-0.30**	-0.30**	-		
(6) DT Machiavellianism	2.84	0.85	-0.43**	-0.44**	-0.28**	-0.32**	0.80**	-	
(7) DT Narcissism	2.53	0.73	-0.20**	-0.38**	-0.07**	-0.07*	0.78**	0.42**	-
(8) DT Psychopathy	2.17	0.75	-0.48**	-0.51**	-0.35**	-0.31**	0.80**	0.44**	0.50**

*N = 1,518. ^∗∗^*p* < 0.01, ^∗^*p* < 0.05*.

Light Triad total scores as well as each of the three Light Triad factors were negatively correlated with all of the factors of the Dark Triad. The Light Triad and its facets were most strongly negatively correlated with Machiavellianism. In a regression analysis in which the unique variance of each member of the Dark Triad was assessed, both Machiavellianism and Psychopathy independently negatively predicted Light Triad scores (*Psychopathy* β = -0.41, *p* < 0.01, *Machiavellianism* β = -0.30, *p* < 0.01), whereas narcissism slightly independently *positively* predicted Light Triad scores (β = 0.15, *p* < 0.01). These findings suggest that the three Light Triad facets are not simply the direct counterparts of the three Dark Triad facets.

#### Light Triad Nomological Network

In order to assess the differing nomological network of the Light and Dark Triad, we examined correlations between the Light Triad and Dark Triad scores and a wide range of variables relating to personality, psychological needs, values, character strengths, psychological defenses, worldview, self-esteem, authenticity, sex, relationships, compassion, morality, life satisfaction, curiosity, and transcendence. We also added columns in gray showing the partial correlation controlling for all of the facets of Agreeableness administered in that particular study and HEXACO Honesty-Humility (in the two studies that administered this scale). Below we will mention each set of findings, grouped conceptually. All of the information about the source of the variables (i.e., which studies they came from) are listed in the associated tables as well as in Table 1.

#### Demographics

Table 5 shows the association between the Light and Dark Triad and relevant demographic information.

**Table 5:** Correlations between demographic variables with light triad, dark triad, and light vs. dark triad.

	*M*	*SD*	Light Triad (Partial)	Light Triad	Dark Triad	Dark Triad (Partial)
Age	35.49	11.45	0.05†	0.22**	-0.26**	-0.15**
Female	52.6%		0.07**	0.20**	-0.28**	-0.21**
Income	-		-	0.09**	0.09**	-
Childhood Income	-		-	0.04	0.06*	-
Childhood Unpredictability	-		0.01	-0.21**	0.12*	0.03
Social Desirability	6.03	2.74	0.11**	0.19**	-0.14**	-0.07*

*Because Income, Childhood Income is an ordinal variable, the reported correlation with this variable was a non-parametric Spearman Rho Rank correlation. N = 1,518; Study 4 did not measure Social Desirability, so N = 1,324 for summary statistics including that variable. Study 1 was the only study that measured childhood predictability, so N = 387 for statistics including that variable. In the partialed column, we controlled for total agreeableness scores across samples. We did not conduct partial correlations with income and childhood income because they are ordinal variables. ^∗∗^p < 0.01, ^∗^p < 0.05, ^†^p < 0.10*.

The Light Triad was negatively correlated with unpredictability in childhood (but not associated with childhood income). The Light Triad was no longer negatively correlated with childhood unpredictability, however, after taking Agreeableness into account. The Light Triad was positively correlated with older age, being female, and higher income. The correlation with being female remained significant, even after controlling for Agreeableness. In contrast, the Dark Triad was negatively associated with age and being female and was positively correlated with childhood unpredictability and weakly positively correlated with income. The Dark Triad remained negatively correlated with age and being female after controlling for Agreeableness.

**Figure 5:**
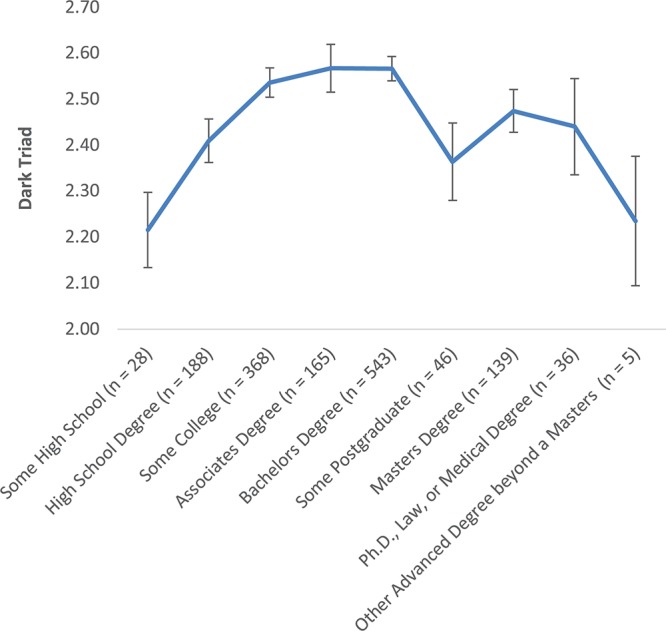
Curvilinear relationship between education and dark triad in the total sample. *N* = 1518.

Because preliminary analyses suggested that the relationships between education and the Light and Dark Triad were non-linear, we conducted one-way ANOVAs followed by polynomial trend analyses. For the Light Triad, there were no differences among education groups, *F*(8,1509) = 0.83, *p* = 0.58. For the Dark Triad, there was a significant overall difference, *F*(8,1509) = 2.82, *p* = 0.004, and a significant quadratic trend, *F*(1,1509) = 16.11, *p* < 0.01, indicating that Dark Triad increased from Some High School to Some College, peaked at Associates and Bachelor’s Degrees, and then decreased with more advanced degrees (see Figure 5).

In terms of socially desirable responding, there was a weak but significant tendency for those scoring higher on the Light Triad to score higher in socially desirable responding (*r* = 0.19, *p* < 0.01, *N* = 1324), and those scoring higher on the Dark Triad to score lower in socially desirable responding (*r* = -0.14, *p* < 0.01, *N* = 1324), and these relationships became weaker (but still remained statistically significant) after controlling for Agreeableness (see Table 5). Nevertheless, since the correlations with socially desirable responding were so weak, we did not expect that socially desirable responding strongly influenced the results reported in this paper.

#### Personality

Table 6 shows the association between the light and Dark Triad and personality, using two different comprehensive Big Five test batteries, as well as the Honesty-Humility facet of the HEXACO model.

**Table 6:** Correlations between personality and light vs. dark triad.

			Light Triad	Dark Triad
Honesty-Humility (HH)^a,b^			0.48**	-0.73**
	HH Sincerity		0.32**	-0.45**
	HH Fairness		0.39**	-0.53**
	HH Greed_Avoidance		0.22**	-0.46**
	HH Modesty		0.49**	-0.71**
Big Five Inventory (BFI)^a,c,d^				
	BFI Open Mindedness		0.29**	-0.04
		BFI Aesthetic Sensitivity	0.28**	-0.13**
		BFI Intellectual Curiosity	0.21**	-0.03
		BFI Creative Imagination	0.23**	0.09*
	BFI Conscientiousness		0.32**	-0.19**
		BFI Organization	0.17**	-0.12**
		BFI Productiveness	0.31**	-0.09**
		BFI Responsibility	0.37**	-0.29**
	BFI Extraversion		0.24**	0.24**
		BFI Social Engagement	0.21**	0.18**
		BFI Assertiveness	0.02	0.40**
		BFI Energy Level	0.39**	0.00
	BFI Agreeableness		0.79**	-0.52**
		BFI Compassion	0.66**	-0.44**
		BFI Respectfulness	0.59**	-0.50**
		BFI Acceptance	0.72**	-0.38**
	BFI Negative Emotionality		-0.30**	0.04
		BFI Anxiety	-0.23**	-0.03
		BFI Depression	-0.32**	0.03
		BFI Emotional Volatility	-0.25**	0.09**
Big Five Aspects Scale (BFAS)^b^				
	BFAS Intellect Openness		0.36**	-0.05
		BFAS Intellect	0.27**	-0.02
		BFAS Openness	0.34**	-0.06
	BFAS Conscientiousness		0.12**	-0.10*
		BFAS Industriousness	0.20**	-0.08*
		BFAS Orderliness	0.01	-0.09*
	BFAS Extraversion		0.26**	0.18**
		BFAS Enthusiasm	0.42**	-0.08*
		BFAS Assertiveness	0.04	0.38**
	BFAS Agreeableness		0.68**	-0.58**
		BFAS Compassion	0.64**	-0.38**
		BFAS Politeness	0.57**	-0.68**
	BFAS Neuroticism		-0.27**	0.13**
		BFAS Withdrawal	-0.23**	0.05
		BFAS Volatility	-0.28**	0.20**

^a^*Measure included in Study 1 (n = 387); ^*b*^Measure included in Study 2 (n = 670); ^*c*^Measure included in Study 3 (n = 267); ^*d*^Measure included in Study 4 (n = 194). ^∗∗^p < 0.01, ^∗^p < 0.05*.

The Light Triad was positively correlated with the Honesty-Humility factor and its associated facets, being particularly associated with the Modesty facet. In a regression model, Modesty, Fairness, and Sincerity independently positively predicted the Light Triad (*Modesty*: β = 0.40, *p* < 0.01; *Fairness*: β = 0.22, *p* < 0.01; *Sincerity:* β = 0.12, *p* < 0.01), whereas Greed Avoidance slightly negatively independently predicted the Light Triad when all of the facets of Honesty-Humility were considered together (β = -0.07, *p* < 0.05). As expected based on the prior literature, the Dark Triad was strongly negatively correlated with Honesty-Humility and its facets (especially the Modest facet). Likewise, in the regression analysis, Modesty made the strongest independent prediction on the Dark Triad (β = -0.53, *p* < 0.01), followed by Fairness (β = -0.25, *p* < 0.01), Sincerity (β = -0.13, *p* < 0.01), and Greed Avoidance (β = -0.10, *p* < 0.01).

In terms of the Big Five Inventory (BFI), the Light Triad was significantly correlated with all five factors of the Big Five, and all of the facets of the Big Five except for Assertiveness. As expected, the Light Triad was strongly correlated with Agreeableness (*r* = 0.79, *p* < 0.01) and all of its facets. In a regression model looking at the BFI Big Five traits, Agreeableness remained the only independent predictor of the Light Triad, and this prediction was high (β = 0.78, *p* < 0.01). In a regression model looking at all of the facets of the BFI, the facets that independently predicted the Light Triad were Acceptance (β = 0.48, *p* < 0.01), Compassion (β = 0.28, *p* < 0.01), Responsibility (β = 0.12, *p* < 0.01), Respectfulness (β = 0.12, *p* < 0.01), and Anxiety (β = 0.07, *p* < 0.01), with Acceptance and Compassion clearly standing out as the strongest independent predictors of the Light Triad.

In contrast, only three of the BFI traits were correlated with the Dark Triad: (higher) Extraversion, (lower) Conscientiousness, and (lower) Agreeableness. At the facet level, the Dark Triad was positively correlated with BFI Social engagement, Assertiveness, Emotional Volatility, and Creative Imagination, and was negatively correlated with Aesthetic Sensitivity, Organization, Productiveness, Responsibility, Compassion, Respectfulness, and Acceptance. In a regression analysis, Assertiveness (β = 0.33, *p* < 0.01), Responsibility (β = -0.28, *p* < 0.01), Acceptance (β = -0.22, *p* < 0.01), Compassion (β = -0.18, *p* < 0.01), Creative Imagination (β = 0.18, *p* < 0.01), Respectfulness (β = -0.18, *p* < 0.01), and Aesthetic Sensitivity (β = -0.10, *p* < 0.01) independently predicted the Dark Triad.

In regards to the BFAS, the Light Triad was correlated with all Big Five traits. Again, the correlation with Agreeableness was the strongest relationship among the Big Five traits (*r* = 0.68, *p* < 0.01). At the facet level of analysis, the Light Triad was correlated with (greater) Intellect, (greater) Openness to Experience, (greater) Industriousness, (greater) Enthusiasm, (greater) Compassion, (greater) Politeness, (less) Withdrawal, and (less) Volatility. In a regression model, the BFAS traits that independently predicted the Light Triad were: *Agreeableness* (β = 0.65, *p* < 0.01), (less) *Conscientiousness* (β = -0.17, *p* < 0.01), *Intellect/Openness* (β = 0.07, *p* < 0.01), and (less) *Neuroticism* (β = -0.15, *p* < 0.01). In a regression analysis looking at the aspects of the BFAS, *Compassion* (β = 0.40, *p* < 0.01), *Politeness* (β = 0.30, *p* < 0.01), (less) *Withdrawal* (β = -0.18, *p* < 0.01), and (less) *Industriousness* (β = -0.15, *p* < 0.01) remained independent predictors of the Light Triad.

In contrast, the Dark Triad was negatively correlated with Conscientiousness and Agreeableness and was positively correlated with Extraversion and Neuroticism. At the aspect level, the Dark Triad was positively correlated with Assertiveness and Volatility, and was negatively correlated with Industriousness, Orderliness, Enthusiasm, Compassion, and Politeness. In a regression, the following aspects independently predicted the Dark Triad: (lower) Politeness (β = -0.54, *p* < 0.01), (higher) Assertiveness (β = 0.37, *p* < 0.01), (higher) Withdrawal (β = 0.19, *p* < 0.01), (lower) Compassion (β = -0.14, *p* < 0.01), (higher) Openness (β = 0.12, *p* < 0.01), and (lower) Intellect (β = -0.10, *p* < 0.01).

#### Psychological Needs and Motives

Table 7 shows the correlations among psychological needs, motives, as well as the Light Triad and Dark Triad.

**Table 7:** Correlations and partial correlations between psychological needs/motives and light vs. dark triad.

		Light Triad (Partial)	Light Triad	Dark Triad	Dark Triad (Partial)
PN Relatedness Aggregate^a^	0.16**	0.53**	-0.25**	0.05
	PN Relatedness Satisfaction	0.26**	0.54**	-0.22**	0.11*
	PN Relatedness Dissatisfaction	-0.02	-0.36**	0.21**	0.02
PN Competence Aggregate^a^	0.09	0.33**	-0.08	0.13*
	PN Competence Satisfaction	0.18**	0.33**	0.01	0.21**
	PN Competence Dissatisfaction	0.04	-0.23**	0.15**	0.00
PN Autonomy Aggregate^a^	0.13*	0.39**	-0.16**	0.10
	PN Autonomy Satisfaction	0.23**	0.39**	-0.07	0.17**
	PN Autonomy Dissatisfaction	-0.01	-0.30**	0.20**	-0.01
Motives				
	UM Power	0.09	-0.17**	0.61**	0.50**
	UM Affiliation	0.15**	0.24***	0.22**	0.24**
	UM Intimacy	0.23**	0.43**	-0.05	0.16**

^a^*Measure included in Study 1 (n = 387). The following subscales were controlled for in the partialed column: HEXACO Sincerity, HEXACO Fairness, HEXACO Greed Avoidance, HEXACO Modesty, BFI Compassion, BFI Respectfulness, BFI Acceptance. ^∗∗^p < 0.01, ^∗^p < 0.05*.

The Light Triad was positively correlated with the satisfaction of the needs for relatedness, competence, and autonomy, and negatively correlated with dissatisfaction of these needs. The correlations between the Light Triad and the satisfaction of these needs remained even after partialing out all of the facets of Agreeableness and HEXACO Honesty-Humility.

In contrast, the Dark Triad was negatively related to satisfaction of Relatedness, and was positively correlated with *dissatisfaction* of relatedness, competence, and autonomy. After partialing out all of the facets of Agreeableness and Honesty-Humility, the Dark Triad was no longer correlated with dissatisfaction of psychological needs, and was even *positively* associated with satisfaction of relatedness, competence, and autonomy. In a regression model looking at the aggregate of all three needs, only (less) Relatedness was an independent predictor of the Dark Triad (β = -0.27, *p* < 0.01). Looking at the satisfaction and dissatisfaction of the psychological needs simultaneously, (less) Relatedness Satisfaction (β = -0.22, *p* < 0.01) and (more) Competence Satisfaction (β = 0.16, *p* < 0.01) were independent predictors of the Dark Triad. There was also a marginal effect of autonomy dissatisfaction on the Dark Triad (β = 0.13, *p* = 0.05).

In terms of core motives, the Light Triad was positively related to the motives for affiliation and intimacy but was negatively related to the power motive. Partialing out all of the facets of Agreeableness and Honesty-Humility, the Light Triad was no longer associated with the power motive but was still significantly positively associated with the motives for affiliation and intimacy. In a regression, all three motives independently predicted the Light Triad (*Intimacy*: β = 0.40, *p* < 0.01; *Power*: β = -0.30, *p* < 0.01; *Affiliation*: β = 0.19, *p* < 0.01).

In contrast, unsurprisingly, the correlation between the Dark Triad and the Power motive was extremely high (*r* = 0.61, *p* < 0.01). The Dark Triad was also positively correlated with the motive for affiliation but was uncorrelated with the motive for intimacy. After controlling for all of the facets of Agreeableness and Honesty-Humility, the Dark Triad was still positively correlated with the motives for power and affiliation, but a positive correlation with intimacy emerged.

#### Values and Character Strengths

Table 8 shows the correlation between the light and Dark Triad and values and character strengths.

**Table 8:** Correlations and partial correlations between values/character strengths and light vs. dark triad.

		Light Triad (Partial)	Light Triad	Dark Triad	Dark Triad (Partial)
**Values^c^**				
	*Self-Transcendence*	0.23**	0.49**	-0.51**	-0.35**
	Universalism Nature	0.07	0.12	-0.18**	-0.13*
	Universalism Concern	0.16*	0.39**	-0.43**	-0.28**
	Universalism Tolerance	0.24**	0.37**	-0.32**	-0.21**
	Benevolence Care	0.08	0.36**	-0.33**	-0.18**
	Benevolence Dependability	0.08	0.26**	-0.26**	-0.13**
	*Self-Enhancement*	-0.35**	-0.52**	0.69**	0.61**
	Achievement	-0.14*	-0.26**	0.56**	0.53**
	Power- Domination	-0.36**	-0.48**	0.60**	0.50**
	Power- Resources	-0.30**	-0.49**	0.53**	0.42**
	*Openness to Change*	-0.02	-0.13**	0.30**	0.23**
	Self-Direction Thought	0.01	-0.09	-0.01	-0.11
	Self-Direction Action	0.02	-0.13*	0.02	-0.07
	Stimulation	-0.01	-0.04	0.36**	0.36**
	Hedonism	-0.04	-0.05	0.28**	0.29**
	*Conservation*	0.09	0.15*	-0.41**	-0.39**
	Security-Personal	-0.07	-0.15*	-0.22**	-0.28**
	Security-Societal	0.01	-0.05	-0.08	-0.12†
	Tradition	-0.03	0.06	-0.01	-0.01
	Conformity-Rules	0.14*	0.12*	-0.36**	-0.37**
	Conformity-Interpersonal	0.15*	0.31**	-0.35**	-0.23**
	Face	-0.10	-0.26**	0.19*	0.11
	Humility	0.25**	0.31**	-0.39**	-0.36**
**VIA Character Strengths^d^**				
	VIA Creativity	0.07	0.09	0.27**	0.31**
	VIA Curiosity	0.20**	0.20**	0.16*	0.21**
	VIA Judgment	0.16*	0.17*	0.16*	0.23**
	VIA Love of Learning	0.14†	0.18*	0.08	0.15**
	VIA Perspective	0.27**	0.41**	-0.04	0.12**
	VIA Bravery	0.04	0.05	0.27**	0.32**
	VIA Perseverance	0.04	0.15*	0.06	0.17*
	VIA Honesty/Authenticity	0.11	0.13†	0.13†	0.14*
	VIA Zest	0.19**	0.37**	0.03	0.21**
	VIA Love	0.25**	0.44**	-0.04	0.13
	VIA Kindness	0.21**	0.51**	-0.13†	0.13
	VIA Social Intelligence	0.19*	0.35**	-0.02	0.14
	VIA Teamwork	0.23**	0.31**	0.09	0.20**
	VIA Fairness	0.10	0.21**	0.11	0.22**
	VIA Leadership	0.11	0.12†	0.28**	0.31**
	VIA Forgiveness	0.18*	0.32**	0.00	0.12
	VIA Humility	0.07	0.17*	-0.04	0.10
	VIA Prudence	-0.03	-0.10	0.08	0.07
	VIA Self Regulation	-0.01	0.10	0.04	0.14
	VIA Appreciation	0.27**	0.32**	0.05	0.14
	VIA Gratitude	0.25**	0.41**	-0.11	0.06
	VIA Hope	0.13	0.25**	0.13†	0.23**
	VIA Humor Playfulness	0.14	0.15*	0.04	0.10
	VIA Spirituality	0.08	0.18*	0.15*	0.23**

*^*c*^Measure included in Study 3 (n = 267); ^*d*^Measure included in Study 4 (n = 194). In Study 3, a more comprehensive battery of the dark triad was administered to participants (see section “Materials and Methods”). The following subscales were controlled for in the partialed column: BFI Compassion, BFI Respectfulness, BFI Acceptance. ^∗∗^p < 0.01, ^∗^p < 0.05, ^†^p < 0.10.*

The Light Triad was strongly positively correlated with the values of Self-Transcendence and Humility and strongly negatively correlated with Self-Enhancement. The Light Triad was also positively correlated with Conservation and negatively correlated with Openness to Change and Face, although these correlations were very small in magnitude. The correlations with (greater) Self-Transcendence, (greater) Humility, and (less) Self-Enhancement remained after partialing out the facets of Agreeableness.

In terms of character strengths, the Light Triad was positively correlated with 18 out of the 24 character strengths, and 11 of these correlations remained significant after controlling for the facets of Agreeableness. In contrast, only six character strengths were positively correlated with the Dark Triad (creativity, curiosity, judgment, bravery, leadership, and spirituality).

#### Defense Styles

Table 9 shows the correlations among the Light and Dark Triad and psychological defenses.

**Table 9:** Correlations and partial correlations between defense styles and light vs. dark triad.

		Light Triad (Partial)	Light Triad	Dark Triad	Dark Triad (Partial)
Defense Style Mature^c^	0.23**	0.37**	-0.05	0.16*
	Mature Suppression	0.21**	0.33**	-0.10	0.06
	Mature Sublimation	0.17**	0.30**	0.02	0.18**
	Mature Humor	0.13*	0.26**	-0.05	0.09
	Mature Anticipation	0.12*	0.15*	-0.01	0.08
Defense Style Neurotic^c^	0.25**	0.39**	0.00	0.18**
	Neurotic Pseudoaltruism	0.25**	0.40**	-0.14*	0.04
	Neurotic Undoing	0.14*	0.09	0.10†	0.12
	Neurotic Idealization	0.06	0.23**	0.14*	0.29**
	Neurotic Reaction Formation	0.22**	0.34**	-0.11†	0.01
Defense Style Immature^c^	-0.09	-0.33**	0.53**	0.46**
	Immature Projection	-0.09	-0.26**	0.20**	0.11
	Immature Passive Aggression	-0.17*	-0.38**	0.35**	0.20**
	Immature Acting Out	0.01	-0.18**	0.45**	0.39**
	Immature Isolation	-0.07	-0.31**	0.39**	0.28**
	Immature Devaluation	-0.05	-0.35**	0.31**	0.16**
	Immature Autistic Fantasy	0.02	-0.25**	0.19**	0.08
	Immature Denial	-0.10	-0.05	0.37**	0.40**
	Immature Displacement	0.06	-0.09	0.14*	0.07
	Immature Dissociation	-0.17**	-0.14*	0.44**	0.48**
	Immature Splitting	-0.06	-0.11†	0.36**	0.38**
	Immature Rationalization	-0.05	0.17**	0.04	0.21**
	Immature_Somatization	0.06	-0.09	0.20**	0.20**

**^*c*^Measure included in Study 3 (n = 267). In Study 3, a more comprehensive battery of the dark triad was administered to participants (see section “Materials and Method”). The following subscales were controlled for in the partialed column: BFI Compassion, BFI Respectfulness, BFI Acceptance. ^∗∗^p < 0.01, ^∗^p < 0.05, ^†^p < 0.10*.*

The Light Triad was positively correlated with mature and with neurotic defense styles, and negatively associated with immature defense styles. The correlations with mature and neurotic defense styles held even after controlling for the facets of Agreeableness. In contrast, the Dark Triad was uncorrelated with mature and neurotic defense styles but showed a strong positive correlation with immature defense styles, which remained even after controlling for the facets of Agreeableness. Interestingly, after controlling for the facets of Agreeableness, the Dark Triad was also positively correlated (although weaker) with mature and neurotic defense styles.

In contrast, the following immature defense styles positively independently predicted the Dark Triad: *Dissociation* (β = 0.30, *p* < 0.01), *Acting Out* (β = 0.29, *p* < 0.01), and *Isolation* (β = 0.26, *p* < 0.01).

#### Worldview

Table 10 shows the correlation among the light and Dark Triad and worldview.

**Table 10:** Correlations and partial correlations between worldview and light vs. dark triad.

	Light Triad (Partial)	Light Triad	Dark Triad	Dark Triad (Partial)
**Cognitive Triad Inventory (CTI)^c^**				
CTI Total	0.15*	0.41**	-0.18**	0.03
CTI Positive View Self	0.10	0.37**	-0.20**	0.02
CTI Positive View World	0.18**	0.43**	-0.24**	-0.07
CTI Positive View Future	0.14*	0.34**	-0.08	0.09
**Beliefs^d^**				
Belief “Humans Are Good”	0.34**	0.54**	-0.26**	-0.10
Belief “I Am Good”	0.08	0.47**	-0.16*	0.07

^c^*Measure included in Study 3 (n = 267); ^*d*^Measure included in Study 4 (n = 194). In Study 3, a more comprehensive battery of the dark triad was administered to participants (see section “Materials and Methods”). The following subscales were controlled for in the partialed column: BFI Compassion, BFI Respectfulness, BFI Acceptance. ^∗∗^p < 0.01, ^∗^p < 0.05*.

The Light Triad was positively correlated with the Cognitive Triad and all of its facets (Positive View of Self, Positive View of the World, and Positive View of the Future). Controlling for the facets of Agreeableness significantly reduced the size of the correlations, but the relationship between the Light Triad and total score, Positive View of World, and Positive View of Future remained statistically significant. The Dark Triad was negatively correlated with the total score, Positive View of Self, and Positive View of World, but was uncorrelated with view of the future. The Dark Triad was no longer correlated with the Cognitive Triad after controlling for the facets of Agreeableness.

Within the Cognitive Triad, Positive View of the World was the only independent predictor of the Light Triad (β = 0.35, *p* < 0.01). Both Positive View of the World (β = -0.30, *p* < 0.01) and Positive View of Self (β = -0.27, *p* < 0.05) negatively independently predicted the Dark Triad, whereas Positive View of Future was a strong *positive* independent predictor of the Dark Triad (β = 0.36, *p* < 0.01). Note that at the zero-order level of analysis, Positive View of Future was uncorrelated with the Dark Triad.

Consistent with these findings, the Light Triad was strongly related to the belief that “Humans are good” and the belief that “I am good.” After controlling for the facets of Agreeableness, the Light Triad was still positively related to the belief that “Humans are good.” In contrast, the Dark Triad was negatively correlated with both beliefs. However, these correlations were not significant after controlling for the facets of Agreeableness.

#### Self-Esteem and Authenticity

Table 11 shows the correlations between Self-Esteem, Authenticity, and the Light vs. Dark Triad.

**Table 11:** Correlations and partial correlations between self-esteem/authenticity and light vs. dark triad.

	Light Triad (Partial)	Light Triad	Dark Triad	Dark Triad (Partial)
**Self Esteem^d^**				
Global Self Esteem	0.00	0.27**	0.18*	0.39**
CSE Family Support	0.10	0.41**	-0.18*	0.03
CSE Competition	-0.07	-0.09	0.29**	0.29**
CSE Appearance	0.01	0.00	0.20**	0.20**
CSE God Love	0.07	0.24**	0.16*	0.28**
CSE Academics	0.15*	0.14*	0.10	0.08
CSE Virtue	0.26**	0.43**	-0.23**	-0.09*
CSE Approval From Others	0.10	0.13†	-0.11	-0.10*
**Weak Sense of Self Total^d^**	0.03	-0.16*	-0.07	-0.23**
**Authenticity Scale^d^**				
Authentic Living	0.30**	0.47**	-0.18*	0.00
Self-Alienation	0.06	-0.30**	-0.01	-0.18*
Accepting External Influence	-0.09	0.12	-0.16*	-0.17*
**Authenticity Inventory^d^**				
Self-Awareness	0.11	0.34**	0.03	0.23**
Unbiased Processing	-0.03	0.20**	-0.03	0.08
Authentic Behavior	0.17*	0.31**	-0.08	-0.01
Relational Authenticity	0.19**	0.48**	-0.14	0.05

*^*d*^Measure included in Study 4 (n = 194). The following subscales were controlled for in the partialed column: BFI Compassion, BFI Respectfulness, BFI Acceptance. ^∗∗^p < 0.01, ^∗^p < 0.05, ^†^p < 0.10*.

The Light Triad was positively correlated with global self-esteem, with self-esteem being especially contingent on the domains of family support, god love, and virtue. After controlling for the facets of Agreeableness, only virtue remained significantly correlated with the Light Triad. The Dark Triad was also positively associated with global self-esteem, but was more contingent on competition, appearance, and god love. The Dark Triad showed a negative correlation with virtue and family support. After controlling for the facets of Agreeableness, the Dark Triad was no longer correlated with family support, but was still positively correlated with global self-Esteem and the contingencies of competition, appearance, god love, and negatively correlated with the contingency of virtue.

In terms of sense of self, the Light Triad was negatively correlated with a weak sense of self, but this correlation was no longer significant after controlling for the facets of Agreeableness. The Dark Triad was uncorrelated with a weak sense of self. However, after controlling for the facets of Agreeableness, the Dark Triad was negatively correlated with a weak sense of self.

In terms of authenticity, the Light Triad was positively correlated with authentic living, self-awareness, unbiased processing, authentic behavior, and relational authenticity, and was negatively correlated with self-alienation. The positive correlation between the Light Triad and authentic living, authentic behavior, and relational authenticity remained even after controlling for the facets of Agreeableness. In contrast, the Dark Triad was negatively correlated with authentic living and accepting external influence and was uncorrelated with the rest of the measures of authenticity. After controlling for the facets of Agreeableness, the Dark Triad was no longer correlated with authentic living, but was still negatively correlated with accepting external influence, and became negatively correlated with self-alienation and positively correlated with self-awareness.

#### Sex, Love, and Relationships

Table 12 shows the correlation between sex, love, relationships, and the light and Dark Triad.

**Table 12:** Correlations and partial correlations between sex/love/relationships and light vs. dark triad.

		Light Triad (Partial)	Light Triad	Dark Triad	Dark Triad (Partial)
**Sociosexuality Index (SOI)^a^**				
	SOI Behavior	0.00	-0.14**	0.29**	0.24**
	SOI Attitude	-0.03	-0.27**	0.32**	0.17**
	SOI Desire	0.03	-0.29**	0.44**	0.22**
	SOI Global	0.00	-0.29**	0.43**	0.25**
**Love Styles^a^**				
	Eros	0.20**	0.36**	-0.12*	0.08
	*Ludus*	-0.11*	-0.45**	0.52**	0.19**
	Storge	0.14**	0.26**	-0.10*	0.02
	*Pragma*	0.04	0.00	0.22**	0.10
	*Mania*	0.06	-0.18**	0.19**	0.00
	Agape	0.24**	0.40**	-0.21**	0.03
**Adult Attachment Scale (AAS)^c^**				
	Anxious Attachment	0.07	-0.17**	0.14*	0.02
	Avoidant Attachment	0.01	-0.40**	0.18**	-0.05

^a^*Measure included in Study 1 (n = 387); ^*c*^Measure included in Study 3 (n = 267). In Study 3, a more comprehensive battery of the dark triad was administered to participants (see section “Materials and Methods”). For SOI and Love Styles, the following subscales were controlled for in the partialed column: HEXACO Sincerity, HEXACO Fairness, HEXACO Greed Avoidance, HEXACO Modesty, BFI Compassion, BFI Respectfulness, BFI Acceptance. For AAS, the following subscales were controlled for in the partialed column: BFI Compassion, BFI Respectfulness, BFI Acceptance. ^∗∗^p < 0.01, ^∗^p < 0.05*.

The Light Triad was negatively associated with sociosexuality and its facets. Controlling for the facets of Agreeableness and Honesty-Humility substantially reduced the correlations to zero. In contrast, the Dark Triad was positively associated with the Sociosexuality Index (SOI) and all of its facets, and these correlations remained significant even after controlling for all of the facets of Agreeableness and Honesty-Humility. In a regression analysis, only SOI Desire (β = -0.20, *p* < 0.01) and SOI Attitude (β = -0.14, *p* < 0.05) were independent predictors of the Light Triad. In contrast both SOI Desire (β = 0.37, *p* < 0.01) and SOI Behavior (β = 0.14, *p* < 0.01) were independent predictors of the Dark Triad. Note that SOI Desire was a particularly strong independent predictor of the Dark Triad.

In terms of love styles, the Light Triad was positively correlated with Eros (romantic), Storge (friendship), and Agape (love for all), and negatively correlated with *Ludus* (game playing). All the significant correlations (except for the negative association with *Mania*) remained significant after controlling for the facets of Agreeableness and Honesty-Humility. In a regression, *Agape* (β = 0.26, *p* < 0.01), *Storge* (β = 0.14, *p* < 0.01), and *Eros* (β = 0.13, *p* < 0.01) were positive independent predictors of the Light Triad, whereas *Ludus* (β = -0.27, *p* < 0.01) and *Mania* (β = -0.26, *p* < 0.05) were negative independent predictors of the Light Triad.

In contrast, the Dark Triad was strongly positively correlated with *Ludus*, followed by positive correlations with *Pragma* and *Mania*, and a negative correlation with Agape. The only correlation with the Dark Triad that remained significant after controlling for the facets of Agreeableness and Honesty-Humility was *Ludus*. Similarly, in a regression analysis, *Ludus* was a very strong positive independent predictor of the Dark Triad (β = 0.47, *p* < 0.01), but *Mania* (β = 0.16, *p* < 0.05) and *Pragma* (β = 0.12, *p* < 0.05) also independently predicted the Dark Triad.

In terms of adult attachment, the Light Triad was negatively correlated with both an anxious and avoidant attachment style. However, these correlations were no longer significant after controlling for the facets of Agreeableness. In a regression, Avoidance was a strong negative predictor of the Light Triad (β = -0.48, *p* < 0.05), whereas Anxious Attachment was a borderline *positive* independent predictor of the Light Triad (β = 0.14, *p* = 0.06).

In contrast, the Dark Triad was positively correlated with both an anxious and avoidant attachment style, although these correlations were no longer significant after controlling for the facets of Agreeableness. In a regression, the only independent predictor of the Dark Triad was Avoidant Attachment (β = 0.16, *p* < 0.05).

#### Empathy, Compassion and Interpersonal Style

Table 13 shows the correlation between Empathy, Compassion, Interpersonal Style, and the Light vs. Dark Triad.

**Table 13:** Correlations and partial correlations between empathy/compassion/interpersonal style and light vs. dark triad.

		Light Triad (Partial)	Light Triad	Dark Triad	Dark Triad (Partial)
**DPES Compassion^a^**	0.31**	0.67**	-0.37**	0.11*
**Empathy Total^b^**	0.26**	0.56**	-0.27**	0.09
Cognitive Empathy	0.23**	0.45**	-0.14**	0.12**
Affective Empathy	0.21**	0.55**	-0.34**	0.03
**Interpersonal Guilt Scale^c^**				
Guilt Total	0.09	0.15*	-0.03	0.05
Survival Guilt	0.23**	0.29**	-0.27**	-0.18**
Separation Guilt	0.06	0.26**	-0.10†	0.06
Omnipotence Guilt	0.16**	0.35**	-0.17**	0.03
Self-Hate Guilt	-0.11	-0.34**	0.32**	0.18**
**Quiet Ego Scale Total^a^**	0.27**	0.66**	-0.32**	0.14*
Detached Awareness	-0.09	0.23**	-0.14**	0.12*
Inclusive Identity	0.23**	0.44**	-0.07	0.14*
Perspective Taking	0.25**	0.62**	-0.39**	-0.02
Personal Growth	0.33**	0.39**	-0.07	0.16**

*^*a*^Measure included in Study 1 (*n* = 387); ^*b*^Measure included in Study 2 (*n* = 670); ^*c*^Measure included in Study 3 (n = 267). In Study 3, a more comprehensive battery of the dark triad was administered to participants (see section “Materials and Methods”). For DPES Compassion and the Quiet Ego Scale, the following subscales were controlled for in the partialed column: HEXACO Sincerity, HEXACO Fairness, HEXACO Greed Avoidance, HEXACO Modesty, BFI Compassion, BFI Respectfulness, BFI Acceptance. For Empathy, the following subscales were controlled for in the partialed column: HEXACO Sincerity, HEXACO Fairness, HEXACO Greed Avoidance, HEXACO Modesty, BFAS Compassion, BFAS Politeness. For Interpersonal Guilt, the following subscales were controlled for in the partialed column: BFI Compassion, BFI Respectfulness, BFI Acceptance. ^∗∗^p < 0.01, ^∗^p < 0.05*.

Consistent with the personality findings, the Light Triad was strongly positively correlated with both Compassion and Empathy, whereas the Dark Triad was negatively correlated with Compassion and Empathy. The Light Triad remained significantly correlated with Compassion and Empathy even after for controlling for the facets of Agreeableness and Honesty-Humility. In a regression, both *Affective Empathy* (β = 0.44, *p* < 0.01) and *Cognitive Empathy* (β = 0.18, *p* < 0.01) independently predicted the Light Triad.

In contrast, the Dark Triad was negatively correlated with Compassion and Empathy. However, after controlling for the facets of Agreeableness and Honesty-Humility, the Dark Triad was *positively* correlated with Compassion and Empathy. In a regression, *Affective Empathy* was a strong negative independent predictor of the Dark Triad (β = -0.40, *p* < 0.01), whereas *Cognitive Empathy* was a slight but significant *positive* independent predictor of the Dark Triad (β = 0.10, *p* < 0.05).

The Light Triad was positively associated with all of the facets of Interpersonal Guilt except for Self-Hate Guilt, which showed a negative association with the Light Triad. Even after controlling for the facets of Agreeableness, the Light Triad was still correlated with Survival Guilt and Omnipotence Guilt. In contrast, the Dark Triad was negatively associated with Survival Guilt and Omnipotence Guilt, but was positively associated with Self-Hate Guilt. After controlling for the facets of Agreeableness, the Dark Triad remained negatively correlated with Survivor Guilt and remained positively correlated with Self-Hate Guilt.

In terms of the Quiet Ego, the Light Triad was positively correlated with the Quiet Ego total score and its facets. Note that the correlation with the Quiet Ego total score was particularly strong, about as high as the correlation with Compassion. All of these correlations (except for inclusive identity) remained significant even after controlling for the facets of Agreeableness and Honesty-Humility. In contrast, the Dark Triad was negatively correlated with the Quiet Ego total scores, as well as with Detached Awareness and Perspective Taking, and was uncorrelated with Inclusive Identity and Personal Growth.

#### Selfishness, Aggression, and Moral Judgment

Table 14 shows the correlation between Selfishness, Aggression, Moral Reasoning, and the Light vs. Dark Triad.

**Table 14:** Correlations and partial correlations between selfishness/aggression/moral reasoning and light triad vs. dark triad.

		Light Triad (Partial)	Light Triad	Dark Triad	Dark Triad (Partial)
**Conspicuous Consumption Difference^a^**	-0.10†	-0.28**	0.28**	-0.05
	Conspicuous Consumption	-0.14**	-0.28**	0.36**	-0.10
	Non-conspicuous Consumption	0.00	0.07	-0.02	-0.10
**Reactive-Proactive Aggression Total^b^**	-0.10*	-0.39**	0.53**	0.22**
	Reactive Aggression	-0.10*	-0.27**	0.37**	0.20**
	Proactive Aggression	-0.07	-0.42**	0.57**	0.18**
**Utilitarian Moral Dilemmas Average^b^**	-0.05	-0.21**	0.26**	0.06
**Selfishness Total^b^**	-0.13**	-0.53**	0.69**	0.36**
	Selfishness Egocentric	-0.09*	-0.54**	0.56**	0.23**
	Selfishness Adaptive	-0.10*	-0.39**	0.58**	0.30**
	Selfishness Pathological	-0.13*	-0.49**	0.70**	0.37**
**Dictator Game (Low–High)^b^**	0.15**	0.27**	-0.22**	-0.02

*^*a*^Measure included in Study 1 (n = 387); ^*b*^Measure included in Study 2 (n = 670). For Conspicuous Consumption, the following subscales were controlled for in the partialed column: HEXACO Sincerity, HEXACO Fairness, HEXACO Greed Avoidance, HEXACO Modesty, BFI Compassion, BFI Respectfulness, BFI Acceptance. For Reactive-Proactive Aggression, Utilitarian Moral Dilemmas, Selfishness, and the Dictator Game, the following subscales were controlled for in the partialed column: HEXACO Sincerity, HEXACO Fairness, HEXACO Greed Avoidance, HEXACO Modesty, BFAS Compassion, BFAS Politeness. ^∗∗^p < 0.01, ^∗^p < 0.05, ^†^p < 0.10.*

The Light Triad was negatively correlated with Conspicuous Consumption, whereas the Dark Triad was positively correlated with Conspicuous Consumption. The negative correlation between the Light Triad and conspicuous consumption remained even after controlling for the facets of Agreeableness and Honesty-Humility, whereas the correlation between the Dark Triad and Conspicuous Consumption was longer significant after controlling for these facets.

In terms of aggression, the Light Triad was negatively correlated with both Reactive and Proactive Aggression, whereas the Dark Triad was positively correlated with both Reactive and Proactive aggression. The negative correlation between the Light Triad and Reactive Aggression remained significant even after controlling for the facets of Agreeableness and Honesty-Humility, as did the positive correlation between the Dark Triad and both Reactive and Proactive Aggression.

The Light Triad was negatively correlated with Utilitarian Moral Reasoning, whereas the Dark Triad was positively related to Utilitarian Moral Reasoning. After controlling for the facets of Agreeableness and Honesty-Humility, Utilitarian Moral Reasoning was no longer associated with either the Light or Dark Triad.

In terms of Selfishness, the Light Triad was strongly negatively correlated with Selfishness and its facets (Egocentric, Adaptive, and Pathological Selfishness), whereas the Dark Triad was strongly positively correlated with Selfishness and each of its facets, particularly with the Pathological facet of Selfishness (*r* = 0.70, *p* < 0.01). All of these correlations remained significant even after controlling for the facets of Agreeableness and Honesty-Humility.

The Dictator Game task was in line with the Selfishness findings; the Light Triad was positively correlated with donations given in the Dictator Game, whereas the Dark Triad was negatively correlated with donations given in the Dictator Game. The correlation between the Light Triad and donations given remained significant even after controlling for the facets of Agreeableness and Honesty-Humility, whereas the Dark Triad was no longer significantly correlated with the Dictator Game after controlling for these facets.

#### Religion, Spirituality, and Self-Transcendence

A one-way ANOVA found no Dark Triad difference on any of the response options on the religiosity demographic item. For the Light Triad, one-way ANOVAs found a few pairwise differences between the Religious and Spiritual responses and the other responses, but no Light Triad difference between the Secular, None, and Other groups, *F*(2,651) = 0.02, *p* = 0.98. Therefore, we created dummy variables for being Spiritual and Religious and ran a two-way 2 × 2 ANOVA with Light Triad as the outcome. The Spiritual by Religious interaction was not significant [*F*(1,1514) = 1.12, *p* = 0.29], but there were main effects of being Spiritual [*F*(1,1514) = 32.87, *p* < 0.01, *d* = 0.36] and Religious [*F*(1,1514) = 5.96, *p* = 0.015, *d* = 0.26]. Therefore, religious and spiritual people are more likely to also be high scorers on the Light Triad scale.

Table 15 shows the correlation between measures of Spirituality, Self-Transcendence, and the Light vs. Dark Triad.

**Table 15:** Correlations and partial correlations between spirituality/self-transcendence and light vs. dark triad.

		Light Triad (Partial)	Light Triad	Dark Triad	Dark Triad (Partial)
**Spiritual Experience^a^**	0.08*	0.15**	0.02	0.09*
**Varieties of Spiritual Experience**				
Unity Experiences^a^	0.14**	0.16**	0.10*	0.14**
God Experiences^a^	0.10*	0.12**	0.06	0.06
**Death Transcendence^b^**				
DT Mysticism	0.04	-0.01	-0.02	-0.04
DT Religious	0.05	0.08	0.31**	0.33**
DT Nature	0.23**	0.29**	-0.17*	-0.07
DT Biosocial	0.04	0.32**	-0.03	0.14
DT Creative	0.10	0.11	0.26**	0.24**

*^*a*^Measure included in Study 2 (n = 670). ^*a*^Measure included in Study 4 (n = 194). For Had Spiritual Experience, Oneness Experiences, and God Experiences, the following subscales were controlled for in the partialed column: HEXACO Sincerity, HEXACO Fairness, HEXACO Greed Avoidance, HEXACO Modesty, BFAS Compassion, BFAS Politeness. For Mysticism, the following subscales were controlled for in the partialed column: HEXACO Sincerity, HEXACO Fairness, HEXACO Greed Avoidance, HEXACO Modesty, BFI Compassion, BFI Respectfulness, BFI Acceptance. For Death Transcendence, the following subscales were controlled for in the partialed column: BFI Compassion, BFI Respectfulness, BFI Acceptance. ^∗∗^p < 0.01, ^∗^p < 0.05*.

The Light Triad was significantly correlated with having had a Spiritual Experience, and this correlation remained significant after controlling for facets of Agreeableness and Honesty-Humility. The Dark Triad was not correlated with having had a Spiritual Experience, although the Dark Triad was positively correlated with having had a spiritual experience once we controlled for the facets of Agreeableness and Honesty-Humility.

The Light Triad was positively correlated with Oneness Experiences and God Experiences, and these correlations remained significant even after controlling for the facets of Agreeableness and Honesty-Humility. The Dark Triad was also positively correlated with Oneness Experiences, and this correlation remained significant even after controlling for the facets of Agreeableness and Honesty-Humility. The Dark Triad was uncorrelated with God Experiences.

In terms of death transcendence, the Light Triad was positively correlated with nature and biosocial forms of death transcendence. After controlling for the facets of Agreeableness, the Light Triad was correlated with nature but was no longer correlated with the biosocial route to death transcendence. In contrast, the Dark Triad was positively correlated with religious and creative forms of death transcendence, and negatively correlated with nature as a route to death transcendence. The positive correlation between the Dark Triad and both the religious and creative routes to death transcendence remained significant after controlling for the facets of Agreeableness.

#### Curiosity

Table 16 shows the correlation between measures of Curiosity and the Light vs. Dark Triad.

**Table 16:** Correlations among curiosity and light triad, dark triad, and light vs. dark triad.

		Light Triad (Partial)	Light Triad	Dark Triad	Dark Triad (Partial)
**Curiosity^d^**				
CEI Stretching	0.26**	0.34**	0.20**	0.35**
CEI Embracing	0.08*	0.08	0.36**	0.18*
Interest Curiosity	0.19**	0.25**	0.11	0.18*
Deprivation Curiosity	0.23**	0.17*	0.25**	0.24**

^d^*Measure included in Study 4 (n = 194). The following subscales were controlled for in the partialed column: BFI Compassion, BFI Respectfulness, BFI Acceptance. ^∗∗^p < 0.01, ^∗^p < 0.05*.

The Light Triad was positively correlated with Stretching, Interest Curiosity, and Deprivation Curiosity, and all of these correlations remained significant after controlling for the facets of Agreeableness. In contrast, the Dark Triad was positively correlated with Stretching, Embracing, and Deprivation Curiosity, and these correlations remained significant even after controlling for the facets of Agreeableness. In a regression, only Stretching positively predicted the Light Triad (β = 0.46, *p* < 0.01), whereas Embracing negatively predicted the Light Triad (β = -0.19, *p* < 0.05). In contrast, Embracing (β = 0.37, *p* < 0.01) and Deprivation Curiosity (β = 0.22, *p* < 0.01) positively predicted the Dark Triad.

#### Life Satisfaction

The Light Triad was positively correlated with Life Satisfaction (*r* = 0.37, *p* < 0.01), whereas the Dark Triad was negatively correlated with life satisfaction (*r* = -0.11). The correlation between the Light Triad and Life Satisfaction remained significant even after controlling for the facets of BFI Agreeableness and Honesty-Humility (*r* = 0.21, *p* < 0.01), whereas the correlation between the Dark Triad and Life Satisfaction was no longer significant after controlling for these facets (*r* = 0.07, *p* > 05).

## Discussion

Across four studies including a wide range of positive and negative outcomes, the Light Triad Scale (LTS) was found to be a reliable and valid measure of a loving and beneficent orientation toward others. While the Light Triad contrasts with the callous and manipulative orientation of the Dark Triad, the Light Triad was not merely the inverse of the Dark Triad. It appears that at least in terms of personality, the absence of darkness does not necessarily indicate the presence of light. As with the literature on positive and negative emotions (Diener and Emmons, 1984; Watson et al., 1988), there appears to be some degree of independence between the Light and Dark Triad, leaving room for people to have a mix of both light and dark traits.

With that said, the Light Triad diverged from the Dark Triad across numerous outcomes drawn from both the Dark Triad and well-being literatures and tended to show stronger outcomes with self-transcendent and growth-fostering outcomes relative to the Dark Triad. Below, we’ll go into greater detail on the contrasting nomological networks of the Light vs. Dark Triad, thereby painting overall portraits of these two very different profiles of human nature.

### Portraits of the Light vs. Dark Triad

First, we replicated a number of findings in the Dark Triad literature and extended these findings to the Light Triad. For example, it has been found that Dark Triad traits are correlated with *greater childhood unpredictability* (Jonason et al., 2013, 2016), *aggression* (Pailing et al., 2014; Dinic and Wertag, 2018; Knight et al., 2018; Paulhus et al., 2018), *utilitarian moral judgment* (Djeriouat and Tremoliere, 2014), *selfishness, power, money, and sociosexuality* (Jonason et al., 2008; Jonason and Buss, 2012; Lee et al., 2013; Kajonius et al., 2015; Jonason and Ferrell, 2016; Balakrishna et al., 2017), and *immature defense styles* (Richardson and Boag, 2016). We replicated these findings and also found that the Light Triad is significantly correlated with the inverse of these outcomes.

Second, by also investigating a number of growth-fostering and well-being-related outcomes, we could see an overall pattern of findings that paints two very different portraits of humanity. We found that the Dark Triad was positively correlated with being younger, being male, being motivated by power, sex, achievement, and affiliation, having self-enhancement values, immature defense styles, conspicuous consumption, selfishness, and creative work and religious immortality as routes to death transcendence. The Dark Triad was *negatively* correlated with life satisfaction, conscientiousness, agreeableness, self-transcendent values, compassion, empathy, a quiet ego, a belief that humans are good, and a belief that one’s own self is good.

The Dark Triad was not associated with exclusively adverse and transgressive psychosocial outcomes, however, and some of the correlates of the dark triad may be considered adaptive, at least in limited contexts or “dark niches” (Paulhus, 2014). One example is our replication of the well-known link between the Dark Triad and short-term instrumental sociosexuality (Jonason et al., 2008). Researchers have suggested that the Dark Triad may have evolved precisely because of the reproductive benefits it conferred on our distant ancestors (particularly men) with these Dark Triad characteristics (Jonason et al., 2008). Regardless of the veracity of this evolutionary argument, depending on one’s goals, and the compatibility of those goals with one’s desired sexual partners, high sociosexuality is not necessarily an aversive psychosocial outcome.

The Dark Triad also showed positive correlations with a variety of variables that could facilitate one’s more agentic-related goals. For instance, the Dark Triad was positively correlated with utilitarian moral judgment and the VIA strengths of creativity, bravery, and leadership, as well as assertiveness, in addition to motives for power, achievement, and self-enhancement. Also, an unexpected correlation between the Dark Triad and curiosity was found, which was localized primarily to the embracing and deprivation forms of curiosity.

Interestingly, after controlling for Agreeableness and HEXACO Honesty-Humility, the Dark Triad demonstrated *positive* associations with various growth-oriented outcomes (e.g., empathy, compassion, quiet ego, and spiritual experience) that were negatively related to the Dark Triad before these antagonistic traits were partialed out. These findings suggest that the callous and manipulative core of the Dark Triad does not do these individuals many favors. It’s likely that the variance that is leftover once the malevolence-related variance of the Dark Triad is removed is associated with agentic extraversion, which may provide a protective factor for those scoring higher on the Dark Triad. This is in line with recent research on narcissism that explicitly separates the antagonistic and agentic extraversion facets of narcissism in predicting well-being (e.g., Kaufman et al., 2018).

In stark contrast, the overall picture provided by the pattern of correlations with the Light Triad was quite different than the Dark Triad. The Light Triad was associated with being older, being female, less childhood unpredictability, as well as higher levels of religiosity, spirituality, life satisfaction, acceptance of others, belief that others are good, belief that one’s self is good, compassion, empathy, openness to experience, conscientiousness, positive enthusiasm, having a quiet ego, and a belief that one can live on through nature and biosociality (having children) after one’s personal death. It is notable that the correlation between the belief that others are good and the Light Triad remained significant even after controlling for Big Five Agreeableness, suggesting that— as initially expected— this belief may be a particularly unique aspect of the Light Triad. Also note that we found a strong correlation between “Humans are Good” and the belief that “I am Good” (*r* = 0.51, *p* < 0.001, *n* = 194). This correlation might be worthy of further investigation in future studies.

Individuals scoring higher on the LTS also reported more satisfaction with their relationships, competence, and autonomy, and they also reported higher levels of secure attachment style and eros in their relationships. In general, the light triad was related to being primarily motivated by intimacy and self-transcendent values. Many character strengths correlated with the Light Triad, including curiosity, perspective, zest, love, kindness, teamwork, forgiveness, and gratitude. Note that the flavor of curiosity associated with Light Triad (primarily stretching) differed from the flavor of curiosity associated with the Dark Triad (primarily embracing and deprivation). Mature defense styles were also associated with the Light Triad, as were optimistic beliefs about the self, the world, and one’s future, as measured by the Beck’s cognitive triad. Individuals scoring higher on the LTS also reported higher self-esteem, authenticity, and a stronger sense of self.

In general, the Light Triad does not appear to be associated with any obvious downsides, with a few possible exceptions depending on the context. The Light Triad was not associated with assertiveness, and was negatively correlated with the *motives* for achievement and self-enhancement (even though the Light Triad was positively related to productivity and competence). In terms of character strengths, unlike the Dark Triad, the Light Triad was uncorrelated with bravery or assertiveness. Such characteristics may be important for reaching one’s more challenging goals and fully self-actualizing. Additionally, in line with our predictions, the Light Triad was related to greater interpersonal guilt— including survivor, separation, and omnipotence forms of guilt. While it may be adaptive to experience these forms of interpersonal guilt for facilitating relationships and repairing damage in a relationship, these forms of guilt may limit one’s ambitions for fear of succeeding while others remain less successful.

The Light Triad was also correlated with greater “reaction formation,” which consisted of the following items: “If someone mugged me and stole my money, I’d rather he be helped than punished” and “I often find myself being very nice to people who by all rights I should be angry at.” While having such “loving-kindness” even for one’s enemies is conducive to one’s own well-being (see Salzberg, 2017), these attitudes, coupled with greater interpersonal guilt, could make those scoring higher on the Light Triad potentially more open to exploitation and emotional manipulation from those scoring higher on the Dark Triad. Indeed, we believe further investigation of the social interactions between extreme light vs. dark triad scorers would be an interesting future line of research.

Nevertheless, taking all of these patterns together, the Light Triad appears correlated with a greater quality of life overall than the Dark Triad across numerous dimensions of well-being and growth. Again, we’d like to emphasize that no one is *all* Light or Dark Triad, and we each differ in our balance of these traits. Nevertheless, it should also be noted that the average light-dark balance showed a substantial skew toward the light side of personality, and extreme malevolence was rare in the samples we studied. Indeed, research has shown that, in general, people tend to view the ‘true’ self in others as both good and moral (Strohminger et al., 2017). Anne Frank may have been on to something in her quote at the beginning of this paper.

### Limitations

This study was limited in a number of ways. First, in the same tradition of the literature on the Dark Triad, the Light Triad was measured through self-report. While we do not see this as problematic in establishing a new construct, we would like to see more unobtrusive and behavioral measures of both the Light Triad and Dark Triad. For this reason, we included a dictator game that involved the distribution of real money, but more behavioral tasks would provide stronger evidence for the validity of these constructs.

Second, all participants were recruited from paid online survey platforms. While research has shown that the data collected from the platforms we used are generally representative (Buhrmester et al., 2011; Peer et al., 2017), we think a fuller confirmation of the validity of both the Light Triad and Dark Triad would benefit from the investigation of more ecologically valid samples, such as criminals and “saints.” Additionally, further research is required to assess the generalizability of the findings to a wider range of cultures (e.g., non-English speaking countries), as well as races and ethnicities.

Third, construct redundancy is an issue. The same researchers who are not interested in the extra predictive validity of the Dark Triad over and above the inverse Agreeableness and the HEXACO Honesty-Humility facet will likely not be interested in the Light Triad. On the other hand, those conducting more granular research on the Dark Triad may be interested in the differences described in this paper. Additionally, those interested in well-being and positive mental processes more generally may be interested in the Light Triad. This study took the debate about whether the Dark Triad provides additional explanatory power seriously, controlling for these traits in various analyses. We found that many of the stronger first-order correlations with the Light Triad remained significant, though at a much smaller effect size, demonstrating the added predictive validity offered by the Light Triad. Also notably, Honesty-Humility was more strongly correlated with the inverse of the Dark Triad than with the Light Triad, while the Light Triad was more strongly correlated with Agreeableness than with Honesty-Humility, suggesting further divergence between these two constructs.

### Future Directions

There are several future directions for research on the Light Triad. Most pressingly, further studies should replicate our findings demonstrating that the Light Triad Scale (LTS) provides useful information over and above the inverse of existing measures of the Dark Triad, Big Five Agreeableness, and the HEXACO Honesty-Humility facet.

Second, as noted above, further research on this topic might benefit from a greater focus on behavioral outcomes, demonstrating that these measures predict differences in behavior between predominantly Light Triad individuals as opposed to predominantly Dark Triad individuals. We believe that the workplace might be a particularly interesting context to explore the effects of Dark Triad and Light Triad individuals on teams, and their relative effects on levels of satisfaction and performance.

Third, research could be done on the occupations and life outcomes associated with the Light vs. Dark triad. Some research has found that individuals with Dark Triad traits are often skilled at climbing organizational hierarchies and negatively impact those around them (Mathieu et al., 2014). What kinds of occupations are most attractive to Light Triad individuals?

Fourth, there is also the question of intervention. Is it possible to enhance Light Triad characteristics? In the current investigation, we found a strong link between the Light Triad and the four main characteristics of a quiet ego: perspective-taking, inclusive identity, detached awareness, and growth-mindedness. Researchers are developing exercises to enhance these characteristics (e.g., Wayment and Bauer, 2017), and it’s an interesting question whether such interventions would also have an effect on Light Triad scores. We also found some evidence that experiences of unity, or self-transcendent experiences (STEs; Yaden et al., 2017a), are positively (though less strongly) correlated with the Light Triad. This raises the possibility that certain kinds of experiences could potentially influence these personality traits. While this is unknown, we believe this would be an exciting area of further study.

Fifth, there is the question of framing. In general, research on this topic ought to be a largely a descriptive endeavor. While we have attempted to be balanced in the foregoing discussion, there is little doubt that we believe that Light Triad individuals are more enjoyable to be around and likely exert a more positive net effect on the world. We acknowledge, however, that it is not our place to moralize these two sub-clinical, interpersonal orientations. Future research should bear this descriptive imperative in mind, and researchers may prefer alternative frameworks to describe the nomological network of these two interpersonal orientations. One alternative framework that is popular within the Dark Triad literature is life history strategy, which employs more neutral labels such as “fast” vs. “slow,” rather than our framing of “adverse” vs. “growth-oriented” (e.g., Jonason et al., 2012b). Therefore, we acknowledge that the overall patterns of results could be interpreted within multiple frameworks in psychology.

Sixth, while the focus of this paper was on the suite of traits that comprise the dark vs. light triad, future research is needed on the differential prediction of the three facets of the LTS: Kantianism, Humanism, and Faith in Humanity. Until such validation and/or further scale development is done, we recommend that researchers focus on the total score of the LTS, as the current studies showed that overall, the LTS is a brief, reliable, and valid measure of an important core of positive traits.

Nevertheless, the current version of the LTS included in these investigations should be viewed as a first-draft, and further studies on a wider range of cultures and over longer stretches of time will have to be conducted to improve the generalizability, reliability, stability, and validity of the Light Triad. Also, while the brevity of the LTS has its advantages, it might not be sufficient to explore the breadth of the Light Triad facets that we discovered. In future work, it might be helpful to go back to a larger pool of items and construct a longer measure.

Finally, just as the scope of dark traits has recently increased beyond the boundaries of the Dark Triad (see Moshagen et al., 2018; Paulhus et al., 2018), the scope of the Light Triad may have to eventually be broadened to include further facets of the positive personality. Since our method of constructing the Light Triad Scale (LTS) was based on a consideration of the conceptual contrast to the Dark Triad, we acknowledge that there could be additional aspects of human beneficence that are not captured by the LTS. Ultimately, a combination of top-down and bottom-approaches will be useful to derive the full breadth of facets that comprise the light personality or the “light character” (Cloninger and Zohar, 2011; Meindl et al., 2015; Garcia and Rosenberg, 2016).

While informing other empirical approaches to studying the moral character, we hope our conceptualization of the Light Triad can also inform a number of philosophical discussions of virtuous character and moral behavior (for a psychology-friendly review of this expansive philosophical literature, see Miller, 2013), as well as more specific philosophical discussions of certain drawbacks to such a temperament, as in Wolf’s (1982) notion of “moral saints” and Schwitzgebel’s (2014) distinction between “jerks” and “sweethearts.”

## Conclusion

In order to research the motivations and behaviors associated with the Dark Triad, we have found it useful to recall someone from our personal lives who fits these characteristics – and the same can be said of the Light Triad. For most people, calling to mind an “everyday psychopath” from one’s own life is about as easy as doing so with an “everyday saint.” We suspect that the reader can easily generate examples of both types of people from his or her personal life – as well as prominent individuals in the public arena. As an emerging research literature has shown, there is little doubt that individuals with Dark Triad traits tend to cause substantial interpersonal, organizational, and institutional harm, and thus warrant research attention. However, we believe that the light side of personality is also worth understanding. We hope the current investigation stimulates further research on the good that those with Light Triad characteristics create in the world.

## Data Availability

The datasets generated for this study are available on request to the corresponding author.

## Ethics Statement

This study was carried out in accordance with the recommendations of the University of Pennsylvania Department of Psychology IRB committee with written informed consent from all subjects. All subjects gave written informed consent in accordance with the Declaration of Helsinki. The protocol was approved by the University of Pennsylvania Department of Psychology IRB Committee.

## Author Contributions

SK and DY developed the study concept. SK, DY, and EH designed the experiments and contributed to the creation of the Light Triad Scale (LTS). DY set up the experiments. EH ran the experiments and cleaned the data. SK and ET conducted the analyses. SK wrote the initial draft of the manuscript. DY, EH, and ET provided edits on the manuscript.

## Conflict of Interest Statement

The authors declare that the research was conducted in the absence of any commercial or financial relationships that could be construed as a potential conflict of interest.
