# Supplementary material for: The Light vs. Dark Triad of Personality: Contrasting Two Very Different Profiles of Human Nature
Source: Front Psychol. 2019 Mar 12;10:467. doi: 10.3389/fpsyg.2019.00467 (PMC6423069; doi:10.3389/fpsyg.2019.00467)
Supplement: Supplementary file 1 [file Table_1.docx]

**Supplemental Materials**

*Further information about the measures used in this Study*

**Demographics**

***Annual Income.*** Participants were asked to report how much they earned, before taxes and other deductions, during the past 12 months on a nine-point scale, ranging from “less than $5,000” to “100,000 and greater.”

***Childhood Income.*** Participants were asked to report their family’s financial situation when they were a child on a seven-point scale, ranging from “very poor” to “very rich.”

***Education.*** Participants were asked to report their education level on a nine-point scale, ranging from “completed some high school” to “other advanced degree beyond a Master’s degree.”

***Unpredictability of Childhood.*** We assessed this using eight items, three of which had been developed for use in a prior research study (Mittal et al., 2015), and five more were created for inclusion in a different study to better and more reliably measure the underlying construct of an Unpredictable Childhood Environment (Young et al., 2018). Example items include, “My parents frequently had arguments or fights with each other or other people in my childhood” and “Things were often chaotic in my house.” Respondents endorsed their agreement with these statements using a five-point scale, with options ranging from “disagree strongly” to “agree strongly.”

***Social Desirability.*** The original 33-item Marlowe-Crowne Social Desirability Scale (M-C SDS, Crowne & Marlowe, 1960) is widely used to assess and control for response bias in self-report research. Strahan and Gerbasi (1972) validated a 13-item version employed in our studies. Respondents were asked to answer “true” or “false” to a series of statements that clearly are or are not “socially desirable.” Example items include, “No matter who I’m talking to, I’m always a good listener” and “I have never been irked when people expressed ideas very different from my own.” A Social Desirability score between 0 and 13 was then calculated to assess whether someone was responding truthfully or was misrepresenting him/herself in order to manage self-presentation.

**Dark Triad Measures**

***The Dark Triad of Personality (SD3- Short; Jones & Paulhus, 2014)*** is a 27-item self-report questionnaire that measures Dark Triad traits, divided into three nine-item subscales: *Machiavellianism* (i.e., “It’s not wise to tell your secrets”), *narcissism* (i.e., “People see me as a natural leader”), and *psychopathy*, (i.e., “I like to get revenge on authorities”). In their study comparing two brief Dark Triad measures, Maples, Lamkin, and Miller (2014) conclude that “Overall, if a short measure of the DT is required, the SD3 yields data that are more consistent with these constructs as they are measured using more established and validated measures” (p. 326). Participants were asked to rate their agreement with these self-reflective statements on a five-point scale, with options ranging from “disagree strongly to “agree strongly”.

***The Psychopathic Personality Inventory – Short Form (PPI-SF;*** Tonnaer et al., 2013) is a 56-item version of the original 187-item PPI (Lilienfeld & Hess, 2001). Its eight-item Machiavellianism-Egocentricity facet was administered in this study in order to measure the Dark Triad trait of Machiavellianism. An example item includes, “I sometimes try to get others to ‘bend the rules’ for me if I can’t change them any other way.” Respondents were asked to decide to what extent each item was true or false as applied to them, selecting from “false,” “mostly false,” “mostly true,” and “true.”

***The Triarchic Personality Measure (TriPM; Patrick, 2010)*** is used to assess the Dark Triad trait of Psychopathy. It is a 58-item self-report measure that proposes three phenotypic components: *boldness* (i.e., “If I really wanted to, I could convince most people of just about anything”), *meanness* (i.e., “I get a kick out of startling or scaring other people”), and *disinhibition* (i.e., “I generally prefer to act first and think later”). Respondents were asked to read each statement and indicate whether it is “true,” “somewhat true,” “somewhat false,” or “false” in best describing them.

***The Five-Factor Narcissism Inventory-Short Form (FFNI-SF) is*** the 60-item short form of the original Five-Factor Narcissism Inventory (FFNI; Glover, Miller, Lynam, Crego, & Widiger, 2012), which is a 148-item self-report inventory of 15 traits designed to assess the basic elements of narcissism from the perspective of a five-factor model. The FFNI assesses both *vulnerable* (i.e., cynicism/distrust, need for admiration, reactive anger, and shame) and *grandiose* (i.e., acclaim seeking, arrogance, authoritativeness, entitlement, exhibitionism, exploitativeness, grandiose fantasies, indifference, lack of empathy, manipulativeness, and thrill-seeking) variants of narcissism. Example items include “I am extremely ambitious” and “I hate being criticized so much that I can’t control my temper when it happens”. Respondents were asked to use a five-point scale to select the answer that best corresponds with their agreement to each statement, ranging from “disagree strongly" to "agree strongly".

**Personality**

***The HEXACO Personality Inventory-Revised-Honesty Humility (HEXACO-60, Ashton & Lee, 2009)*** is one of six subscales comprising the 60-item HEXACO personality inventory. It contains 10 items, which are divided into four facets: *sincerity* (e.g., “I wouldn’t pretend to like someone just to get that person to do favors for me”), *fairness* (e.g., “I would never accept a bribe, even if it were very large”), *greed-avoidance* (e.g., “Having a lot of money is not especially important to me”), and *modesty* (e.g., “I want people to know that I am an important person of high status”; reverse coded). Respondents were asked to report their level of agreement with each item on a five-point scale, ranging from “disagree strongly” to “agree strongly.”

***The Big Five Inventory—2 (BFI-2; Soto & John, 2017)*** is a 60-item scale that measures three more-specific facets for each of the Big Five domains of personality: Extraversion (*sociability, assertiveness, energy level*)*,* Agreeableness (*compassion, respectfulness, trust*)*,* Conscientiousness (*organization, productiveness, responsibility*)*,* Negative Emotionality (*anxiety, depression, emotional volatility*)*,* and Open-Mindedness (*intellectual curiosity, aesthetic sensitivity, creative imagination*). Respondents were asked to report their level of agreement with each item on a five-point scale, ranging from “disagree strongly” to “agree strongly.”

***The Big Five Aspect Scales (BFAS; DeYoung et al., 2007)*** is a 100-item scale that measures two aspects of each Big Five factor: Neuroticism (*volatility, withdrawal*), Agreeableness (*compassion, politeness*), Conscientiousness (*industriousness, orderliness*), Extraversion (*enthusiasm, assertiveness*), and Openness/Intellect (*intellect, openness*)*.* Example items include, “I worry about things” and “I see beauty in things that others might not notice.” Respondents were asked to report their level of agreement with each item on a five-point scale, ranging from “strongly disagree” to “strongly agree.”

**Psychological Needs & Motives**

***The Balanced Measure of Psychological Needs Scale (BMPN, Sheldon & Hilpert, 2012)*** is an 18-item questionnaire, containing three six-item subscales to evaluate the degree of satisfaction and dissatisfaction of three basic psychological needs: *autonomy* (e.g., “My choices expressed my ‘true self’”), *competence* (e.g., “I took on and mastered hard challenges”)*, and relatedness* (e.g., “I felt close and connected with other people who are important to me”)*.* Respondents were asked to report their level of agreement with each item on a five-point scale, ranging from “disagree strongly” to “agree strongly.”

***The Unified Motives Scales (UMS, Schönbrodt & Gerstenberg, 2012)*** is a 40-item measure of four explicit motives: *achievement* (e.g., “I enjoy personally doing things better than they have been done before”), *power* (e.g., “I try to control others rather than permit them to control me”), *affiliation* (e.g., “I feel a rush of energy when I get to know new people”), and *intimacy* (e.g., “I like to fully immerse myself in a relationship”). Due to an administration error, we excluded all data for the achievement subscale. The scale also includes a measure of general avoidance/fear, but those items were not included in the current study. Respondents were asked to report their level of agreement with each item on a five-point scale, ranging from “disagree strongly” to “agree strongly.”

**Values & Character Strengths**

***The Portrait Values Questionnaire-Revised (PVQ-RR, Schwartz et al., 2012)*** consists of 57 items, designed to measure the 19 values that are differentiated in Schwartz et al.’s refined theory of basic values (2012; enumerated below). Each value is represented by a subscale containing three verbal ‘portraits’ of different people, describing a value that is important for the person (e.g., “It is important to her to form her views independently.” Because gendered pronouns are used, participants who identified as female at the beginning of the survey received a version that used “she/her.” Males and those who did not identify with one particular gender or preferred not to respond received a version that used “he/him/his” in the statements. Participants were asked to read each description and to think about how much that person is or is not like them and to indicate their response using a six-point scale, ranging from “not at all like me” to “very much like me.” Combined, the means of the subscales of *universalism-nature,* *universalism-concern*, *universalism-tolerance, benevolence-care, and benevolence-dependability* represent a Self-Transcendence higher-order value factor. Combined, the means of the subscales of *achievement*, *power-dominance,* and *power-resources* represent a Self-Enhancement higher-order value factor. Combined, the means of the subscales of *self-direction-thought, self-direction-action, stimulation*, and *hedonism* represent an Openness to Change higher-order value factor. Combined, the means of the subscales of *security-personal, security-societal, tradition, conformity-rules*, and *conformity-interpersonal* represent a Conservation higher-order value factor. *Face* and *humility*, which didn’t fit cleanly into any of the other categories, were analyzed separately. For all correlational analysis, centered scores were used to correct for scale bias. For all regression analyses, uncentered scores were used. Both of these statistical procedures were recommended in the scoring and analysis instructions provided by Schwartz (personal correspondence).

***The Values in Action (VIA) Brief Strengths Test (Peterson & Seligman, 2004)*** is a 24–item self–report questionnaire that measures the degree to which respondents endorse personal character strengths. There is a total of 24 strengths of character in the VIA Classification, upon which this scale is based. The Brief Strengths Test contains one item corresponding to each of the Strengths. An example item from the scale is, “Think of actual situations **during the past month** in which you experienced failure or a setback. How frequently did you show HOPE or OPTIMISM in these situations?” Respondents were asked to use **a five-point** scale, ranging from “never/rarely” to “always.” They also had the option to select “not applicable” if they had not encountered a described situation.

**Defense Styles**

***The Defense Style Questionnaire (DSQ; Andrews, Singh, & Bond, 1993)*** is a 40-item questionnaire that measures Defense styles based on the DSM-III-R draft glossary of defense mechanisms (Advisory Committee on Defense Mechanisms, 1986). According to the DSM-III-R (American Psychiatric Association, 1987), defense mechanisms are defined as “patterns of feelings, thoughts, or behaviors that are relatively involuntary and arise in response to perceptions of psychic danger. They are designed to hide or to alleviate the conflicts or stressors that give rise to anxiety (p. 393)”. The DSQ measures possible conscious derivatives of defense mechanisms and has been found to discriminate between psychiatric patients and nonclinical subjects, with patients endorsing more “immature” defense styles (Andrews et al., 1989; Bond et al., 1983). This study used a revised version of the original 72-item DSQ (Andrews et al., 1989). Respondents were asked to report their level of agreement with each item on a five-point scale, ranging from “disagree strongly” to “agree strongly.” The scale includes two items for each of the 20 defense styles that had the best psychometric properties and ability to discriminate between nonclinical subjects and those diagnosed with anxiety in prior research.

**Worldview**

***The Cognitive Triad Inventory (CTI; Beckham et al., 1986)*** is a 30-item questionnaire that measures the Cognitive Triad” which Aaron Beck argues is an important predictor of depression (e.g., Beck, Rush, Shaw, & Emery, 1979). The cognitive triad consists of negative perceptions of one’s *self* (e.g., “I am a failure”), their *world* (e.g., “The world is a very hostile place”), and their *future* (e.g., “Things will work out for me in the future”). Respondents were asked to report their level of agreement with each item on a five-point scale, ranging from “disagree strongly” to “agree strongly.”

***Beliefs****.* In order to measure Belief in Human Goodness and Belief in Self Goodness we asked participants to rate their agreement with the statements “Humans are good” and “I am good” on a five-point scale, ranging from “disagree strongly” to “agree strongly.”

**Self-Esteem & Authenticity**

***The Rosenberg Self-Esteem Scale (RSES; Rosenberg, 1965)*** is a 10-item index of global Self-esteem that measures both positive and negative feelings about the self. Sample items include, “I feel I am a person of worth, at least on an equal basis of others” and “I feel that I have a number of good qualities.” Respondents were asked to report their level of agreement with each item on a five-point scale, ranging from “disagree strongly” to “agree strongly.”

***The Contingencies of Self Worth Scale (CSW, Crocker, Luhtanen, Cooper, & Bouvrette, 2003)*** is a 35-item scale assessing seven contingencies shown to be important internal and external sources of self-esteem. The subscales include *family support* (e.g., “Knowing that my family members love me makes me feel good about myself”), *competition* (e.g., “I feel worthwhile when I perform better than others on a task or skill”), *appearance* (e.g., “When I think I look attractive, I feel good about myself”), *God’s love* (e.g., “My self-esteem goes up when I feel that God loves me”), *academic competence* (e.g., “Doing well in school gives me a sense of self- respect”), *virtue* (e.g., “I couldn’t respect myself if I didn’t live up to a moral code”), and *approval from others* (e.g., “I can’t respect myself if others don’t respect me”). Respondents were asked to report their level of agreement with each item on a five-point scale, ranging from “disagree strongly” to “agree strongly.”

***The Sense of Self Scale (SOSS; Flury & Ickes, 2007)*** is a 12-item measure that assesses the extent to which one has a weak versus strong Sense of Self. While the scale is unidimensional, it has items relating to four components of a weak sense of self: (1) *Tendency to confuse one’s feelings, thoughts, and perspectives with those of others* (e.g., “I’m not sure that I can understand or put much trust in my thoughts and feelings); (2) *Lack of understanding of oneself* (e.g., “Who am I? is a question that I ask myself a lot”); (3) *Sudden shifts in feelings, opinions, and values* (e.g., “I wish I were more consistent in my feelings”); and (4) *Feeling of a tenuous existence* (e.g., “I often think how fragile my existence is”). Respondents were asked to report their level of agreement with each item on a five-point scale, ranging from “disagree strongly” to “agree strongly”. The SOSS predicts splitting, identity impairment, borderline symptomatology, low self-esteem, low individuation, and engaging in behaviors indicative of a weak sense of self (Flury & Ickes, 2007).

***The Authenticity Scale (TAS; Wood et al., 2008)*** is a 12-item Authenticity scale that has three subscales: *authentic living* (e.g., “I live in accordance with my values and beliefs”), *alienation from the self* (e.g., “I feel out of touch with the ‘real me’”; reverse coded), and *accepting external influenc*e (e.g., “I always feel I need to do what others expect me to do”; reverse coded). Respondents were asked to report their level of agreement with each item on a five-point scale, ranging from “disagree strongly” to “agree strongly.”

***The Authenticity Inventory*** (AI-3; Kernis & Goldman, 2006) is a 45-item measure “conceptually designed to assess the unimpeded operation of one’s True Self (or core self) in one’s daily enterprise.” It contains four subscales: *awareness* (e.g., “For better or for worse I am aware of who I truly am”), *unbiased processing* (e.g., “I am very uncomfortable objectively considering my limitations and shortcomings”; reverse scored), *behavior* (e.g., “I frequently pretend to enjoy something when in actuality I really don’t”), and *relational orientation* (e.g., “I want people with whom I am close to understand my strengths”). Respondents were asked to indicate their agreement with each statement, using a five-point scale, with options ranging from “disagree strongly” to “agree strongly.”

**Sex, Love, & Relationships**

***The Revised Sociosexual Orientation Inventory (SOI-R, Penke & Asendorpf, 2008)*** is a nine-item self-report questionnaire designed to measure individual differences in the tendency to engage in sexual relationships without deeper emotional commitment. This scale is divided into three facets measured by the inventory: *behavior*—in terms of number of casual and changing sex partners (e.g., “With how many different partners have you had sex within the past 12 months?” with nine answer choices ranging from “0” to “20 or more”), *attitude*—towards uncommitted sex (e.g., “Sex without love is OK”, with nine answer choices ranging from “1-strongly disagree” to 9-strongly agree”), and *desire*—for people not in a romantic relationship (“How often do you have fantasies about having sex with someone you are not in a committed romantic relationship with?”, with nine answer choices, ranging from “1-never” to “9 -at least once a day”).

***The Love Attitudes Scale (LAS, Hendrick & Hendrick, 1986)*** is a 42-item questionnaire designed to measure Love Attitudes. The questionnaire combines attitudes toward one’s current (or else, recent or hypothetical) partner with attitudes about love in general. The scale is broken into six subscales (seven items each) that represent a different love style: *eros*—passionate love (e.g., “My lover and I were attracted to each other immediately after we first met”), *ludus*—game-playing love (e.g., I have sometimes had to keep two of my lovers from finding out about each other”), *storge*—friendship love (e.g., “I expect to always be friends with the one I love”), *pragma*—practical love (e.g., “It is best to love someone with a similar background”), *mania*—possessive, dependent love (e.g., “When I am in love, I have trouble concentrating on anything else”), and *agape*—altruistic love (e.g., “I would rather suffer myself than let my lover suffer”). Respondents were asked to report their level of agreement with each item on a five-point scale, ranging from “disagree strongly” to “agree strongly.”

***The Adult Attachment Scale-- Revised (AAS; Collins, 1996)*** is an 18-item scale that measures the attachment styles of adults. Consistent with Fraley & Spieker (2003), we computed two attachment styles: *anxious* (the extent to which a person is worried about being rejected or unloved; e.g., “I often worry that romantic partners won’t want to stay with me”, “I often worry that romantic partners don’t really love me”) and *avoidant* (the extent to which a person avoids intimacy and feels he/she can depend on others to be available when needed; e.g., “Romantic partners often want me to be emotionally closer than I feel comfortable being”, “I find it difficult to trust others completely”). According to Fraley & Spieker (2003), the extent to which a person scores low on both of these dimensions is the extent to which the person is securely attached. Respondents were asked to report their level of agreement with each item on a five-point scale, ranging from “disagree strongly” to “agree strongly”.

**Empathy, Compassion, & Interpersonal Style**

***The Dispositional Positive Emotion Scales Questionnaire-Compassion (DPES, Shiota, Keltner, & John, 2006)***is one of seven subscales contained in the 38-item DPES self-report instrument. The five-item *compassion* subscale measures one’s dispositional tendency to feel compassion toward people in general. An example item is, “It’s important to take care of people who are vulnerable.” Respondents were asked to report their level of agreement with each item on a seven-point scale, ranging from “disagree strongly” to “strongly agree.”

***The Cognitive, Affective, and Somatic Empathy Scales (CASES,*** Raine & Chen, 2018) is a 30-item measure containing three subscales. We administered the 10-item *cognitive empathy* subscale, which refers to the capacity to cognitively understand how others feel (e.g., “When a friend is teased, I understand why they get upset”) and the 10-item *affective empathy* subscale, which refers to the capacity to experience the emotions of how others feel (e.g., “I feel on edge when I see someone looking scared in a movie”). Respondents were asked to indicate how much they endorse each statement or how applicable it is to them, by selecting “rarely,” “sometimes,” or “often.”

***The Interpersonal Guilt Questionnaire (ICQ; O’Connor et al. 1997)*** is a 67-item scale that assesses four types of Guilt: *survivor* (e.g., “I sometimes feel I don’t deserve the happiness I achieved”), *separation* (e.g., “It makes me anxious to be away from home for too long”), *omnipotent responsibility* (e.g., “I worry a lot about the people I love even when they seem to be fine”), and *self-hate* (e.g., “If something bad happens to me I feel I must have deserved it”). Respondents were asked to report their level of agreement with each item on a five-point scale, ranging from “disagree strongly” to “agree strongly.”

***The Quiet Ego Scale (QES; Wayment et al., 2014)*** measures “a self-identity that transcends egoism and identifies with a less defensive, balanced stance toward the self and others.” This 14-item Quiet Ego Scale is comprised of four subscales dedicated to measuring the following well- known psychological characteristics: *detached awareness* (e.g., “I find myself doing things without paying much attention”), *inclusive identity* (e.g., “I feel a connection to all living things”), *perspective taking* (e.g., “Before criticizing somebody, I try to imagine how I would feel if I were in their place”), and *growth* (e.g., “I have the sense that I have developed a lot as a person over time”). Respondents were asked to indicate their agreement with the statements using a five-point scale, ranging from “disagree strongly” to “agree strongly.” Higher scores indicate greater quiet ego characteristics.

**Selfishness, Aggression, & Moral Judgment**

***The Conspicuous Consumption—Extra Money Scale (Lee et al., 2013)*** was constructed for use in a prior research experiment examining the relationships of sex, power, and money to Dark Triad characteristics. Participants were asked to indicate how they would spend an extra $100,000 on 13 items that represent either *conspicuous consumption* (e.g., luxury cars, high-end restaurant meals, etc.) or *non-conspicuous consumption* (e.g., health products, insurance, etc.). The means of the conspicuous items and of the non-conspicuous items were calculated separately. We obtained the final scale scores for this scale by subtracting non-conspicuous scale scores from conspicuous scale scores.

***Reactive-Proactive Aggression Questionnaire (RPQ, Raine et al., 2006)*** is a 23-item, scale that measures the two-factor model of Aggression, with 11 questions examining reactive aggression and 12 questions assessing proactive aggression. Questions ask participants “how often” they have done something, with response options including 0 (never), 1 (sometimes), or 2 (often). An example of a *proactive aggression* item is, “vandalized something for fun,” An example of a *reactive aggression* item is “damaged things because you felt mad.” Although the RPQ was originally developed to assess reactive and proactive aggressive behavior in children, subsequent research has indicated it has clinical relevance for adult populations and that it can distinguish severity levels of aggression (Brugman et al., 2017).

***Utilitarian Moral Dilemmas***. Utilitarian Moral Decision-Making was assessed using three condensed versions of high-conflict personal dilemmas— *crying baby*, *footbridge*, and *sacrifice* (Glenn, Raine, & Schug, 2009). Participants were asked to rate how morally appropriate or inappropriate they found utilitarian actions (ones that are harmful but benefit the greater good) on a seven-point Likert scale with 1 indicating “extremely inappropriate” and 7 indicating “extremely appropriate”.

***The Selfishness Questionnaire (SQ;***Raine & Uhi, 2018) is a 24-item self-report instrument designed to measure Selfish behaviors and attitudes. It is comprised of three subscales: *egocentric* (e.g., “I care for myself much more than I care for others”), *adaptive* (e.g., “At the end of the day, I care mostly for myself, my family, and friends who can help me”), and *pathological* (e.g., “If I’m honest, there are times when I put myself first, even if it’s someone else’s loss”). Respondents were asked to indicate their agreement with each item, choosing from “disagree, “neither agree nor disagree,” or “agree.”

***Dictator Game***. This is an experimental economic task in which participants decide how much, if any, of the money awarded to them by the experimenter they wish to give away to another recipient, without any negative consequences. Similar to Eckel and Grossman’s (1996) version of the dictator game, the described recipient was a charity foundation (*Save the Children*). Participants were informed that they would be given an additional $0.70 for their participation in the study and were asked how much they would be willing to donate to *Save the Children*.

**Religion, Spirituality, & Self-Transcendence**

***Religious Views.*** Participants were asked on a seven-point scale to report their religious views. Possible response options were as follows: 1= religious but not spiritual, 2= spiritual but not religious, 3=secular, 5=religious and spiritual, 6= none, 7=other.

***Spiritual Experience (Yaden & Newberg, in prep)****.* We asked participants the following: “Have you had what you consider to be a spiritual experience? Spiritual experiences are generally considered brief, intense, and vivid subjective experiences involving perceiving an unseen order or connecting to something larger than yourself. People of all belief systems (e.g., secular, spiritual, religious) report having had such experiences.” Participants then rated the extent to which they had a spiritual experience on a five-point scale, that ranging from “definitely not” to “definitely yes”.

***The Varieties Scale (Yaden & Newberg, in prep)****.* This scale is an operationalization of distinctions within William James’s (1902) *The Varieties of Religious Experience.* Participants were asked to indicate the extent to which they have had an experience involving a sense of unity (mystical factor), with items such as “I felt a sense of oneness with all things” or of God/divinity (numinous factor), with items such as “I felt that I encountered the divine.” Participants rated the extent to which they had a Unity or God experience on a seven-point scale, ranging from “strongly disagree” to “strongly agree.”

***The Death Transcendence Scale (DTS;***Hood & Morris, 1983) contains 23 items, based on the premise that "death is transcended through identification with phenomena more enduring than oneself." Items are divided amongst five subscales: *mysticism* (e.g., “I have had an experience in which I felt everything in the world to be part of the same whole”), *religious* (e.g., “I believe in life after death”), *nature* (e.g., “Only nature is forever”), *creative* (e.g., “meaningless work makes for a meaningless life”), and *biosocial* (e.g., “Without children life is incomplete”). VandeCreek and Nye (1993) added three additional items to the biosocial subscale, which we also administered. Thus, respondents were asked to rate their agreement with 26 items total, using a five-point scale, ranging from “disagree strongly” to “agree strongly.”

**Curiosity**

***The Curiosity and Exploration Inventory-II (CEI-II, Kashdan et al., 2009)*** is a 10-item self-report instrument assessing individual differences in the recognition, pursuit, and integration of novel and challenging experiences and information. It contains two factors, with five items devoted to each: *stretching* (e.g., “I am at my best when doing something that is complex or challenging”) and *embracing* (e.g., “I am the type who really enjoys the uncertainty of everyday life”).

***The Epistemic Curiosity Scale (ECS; Litman & Spielberger, 2003)*** is a 10-item instrument developed specifically to assess individual differences in Epistemic Curiosity— the desire for new knowledge. The scale contains items to assess *interest* (I-type) *curiosity*, which is meant to “stimulate pleasurable feelings of situational interest.” An example item is, “I enjoy exploring new ideas.” It also contains items to assess *deprivation* (D-type) *curiosity*, which is meant to “relieve negative affective conditions of feeling deprived of knowledge”. An example item is “I spend hours on a problem because I cannot rest without an answer.” Respondents were asked to indicate their agreement with these statements using a five-point scale, ranging from “disagree strongly”; to “agree strongly”.

**Life Satisfaction**

***The Satisfaction with Life Scale*** (SWLS, Diener et al., 1985) is a self-report instrument of five items answered on a seven-point scale, ranging from “strongly agree” to “strongly disagree,” to assess global life satisfaction. An example item is, “In most ways my life is close to my ideal.”

**Additional Regressions**

**Psychological Needs & Motives**

In a regression model looking at the aggregate of all three needs, *relatedness* (β=.44, p < .001) and *autonomy* (β=.13, p < .001) remained a significant independent predictor of the Light Triad. In a regression model looking at the satisfaction and dissatisfaction of the needs, only *relatedness satisfaction* (β=.42, p < .001) and *autonomy satisfaction* (β=.11, p < .001) were independent predictors of the Light Triad.

In a regression model looking at the aggregate of all three needs, only (less) *relatedness* was an independent predictor of the Dark Triad (β=-.27, p < .001). Looking at the satisfaction and dissatisfaction of the psychological needs simultaneously, (less) *relatedness satisfaction* (β=-.22, p < .001) and (more) *competence satisfaction* (β=.16, p < .001) were independent predictors of the Dark Triad. There was also a marginal effect of *autonomy dissatisfaction* on the Dark Triad (β=.13, p =.05).

In a regression, all three motives independently predicted the Light Triad (*intimacy*: β=.40, p < .001; *power*: β=-.30, p < .001; *affiliation*: β=.19, p < .001).

In a regression analysis, *power* was a strong positive independent predictor of the Dark Triad (β=.62, p <.001), whereas *intimacy* was a negative independent predictor of the Dark Triad (β=-.18, p < .001).

**Values & Character Strengths**

Within Self-Transcendence, the three independent predictors of the Light Triad were *universalism-concern* (β=.27, p < .001), *universalism-tolerance* (β=.25, p < .001), and *benevolence-care* (β=.22, p < .01). Within Self-Enhancement, *achievement* positively independently predicted the Light Triad (β=.31, p < .001), whereas both *power-resources* (β=-.34, p < .001) and *power-dominance* (β=-.24, p < .001) were negative independent predictors of the Light Triad. None of the values within Openness to Change independently predicted the Light Triad. Within Conservation, *conformity-interpersonal* was the only independent predictor of the Light Triad (β=.41, p < .001).

The Dark Triad was strongly positively correlated with Self-Enhancement, and this was uniquely driven by higher endorsement of *stimulation* (β=.29, p < .001) and *hedonism* (β=.17, p < .001). The Dark Triad was also strongly negatively correlated with Self-Transcendence, and this was uniquely driven by less *universalism-concern* (β=-.28, p < .001) and *benevolence-care* (β=-.16, p < .05). The Dark Triad was moderately associated with Conservation, and this was uniquely driven by lower endorsement of *security-personal* (β=-.16, p < .001), *conformity-rules* (β=-.27, p < .001), and *conformity-interpersonal* (β=-.32, p < .001).

The following four character strengths were independently related to the Light Triad: *kindness* (β=.23, p < .01), *teamwork* (β=.17, p < .05), *forgiveness* (β=.21, p < .05), and (less) *bravery* (β=-.16, p < .05). In contrast, the following three character strengths were independently related to the Dark Triad: *leadership* (β=.30, p < .01), *bravery* (β=.26, p < .01), and *creativity* (β=.19, p < .05).

**Defense Styles**

Running a regression analysis of the mature defense styles, *suppression* (β=.24, p < .001), *sublimation* (β=.21 p < .001), and *humor* (β=.17, p < .01) were all significant independent predictors of the Light Triad. In terms of neurotic defense styles, *pseudoaltruism* (β=.35, p < .001), *reaction formation* (β=.25, p < .001), and *idealization* (β=.12, p < .05) were positive independent predictors of the Light Triad, whereas *undoing* (β=-.14, p < .05) was a significant negative predictor of the Light Triad. In terms of immature defense styles, all of the following defense styles negatively independently predicted the Light Triad: *passive aggression* (β=-.25, p < .001), *isolation* (β=-.22, p < .001), *devaluation* (β=-.20, p < .001), *dissociation* (β=-.15, p < .05). and *denial* (β=-.14, p < .05). *rationalization* (β=.16, p < .05) positively independently predicted the Light Triad.

In contrast, the following immature defense styles positively independently predicted the Dark Triad: *dissociation* (β=.30, p < .001), *acting Out* (β=.29, p < .001), and *isolation* (β=.26, p < .001).

**Worldview**

Within the Cognitive Triad, *positive view of the world* was the only independent predictor of the Light Triad (β=.35, p < .001). Both *positive view of the world* (β=-.30, p < .01) and *positive view of self* (β=-.27, p < .05) negatively independently predicted the Dark Triad, whereas *positive view of future* was a strong *positive* independent predictor of the Dark Triad (β=.36, p < .001). Note that at the zero-order level of analysis, Positive View of Future was uncorrelated with the Dark Triad.

**Self-Esteem & Authenticity**

In a regression analysis, *virtue* (β=.30, p < .001) and *family support* (β=.26, p < .001) were positive independent predictors of the Light Triad, whereas *competition* was a negative independent predictor of the Light Triad (β=-.28, p < .001). In contrast, *family support* (β=-.21, p < .001), *virtue* (β=-.20, p < .001), and *approval from others* (β=-.15, p < .001) negatively independently predicted the Dark Triad, whereas *competition* (β=.30, p < .001) and *god love* (β=.26, p < .001) were positive independent predictors of the Dark Triad.

**Sex, Love, & Relationships**

In a regression analysis, only SOI *desire* (β=-.20, p < .01) and SOI *attitude* (β=-.14, p < .05) were independent predictors of the Light Triad. In contrast both SOI *desire* (β=.37, p < .001) and SOI *behavior* (β=.14, p < .01) were independent predictors of the Dark Triad. Note that SOI *desire* was a particularly strong independent predictor of the Dark Triad.

In a regression, *agape* (β=.26, p < .001), *storge* (β=.14, p < .01), and *eros* (β=.13, p < .01) were positive independent predictors of the Light Triad, whereas *ludus* (β=-.27, p < .001) and *mania* (β=-.26, p < .05) were negative independent predictors of the Light Triad.

Similarly, in a regression analysis, *ludus* was a very strong positive independent predictor of the Dark Triad (β=.47, p < .001), but *mania* (β=.16, p < .05) and *Pragma* (β=.12, p < .05) also independently predicted the Dark Triad.

In a regression, *avoidant attachment* was a strong negative predictor of the Light Triad (β=-.48, p < .05), whereas *anxious attachment* was a borderline positive independent predictor of the Light Triad (β=.14, p = .06).

In a regression, the only independent predictor of the Dark Triad was *avoidant attachment* (β=.16, p < .05).

**Empathy, Compassion & Interpersonal Style**

In a regression, both *affective empathy* (β=.44, p < .001) and *cognitive empathy* (β=.18, p < .001) independently predicted the Light Triad.

In a regression, *affective empathy* was a strong negative independent predictor of the Dark Triad (β=-.40, p < .001), whereas *cognitive empathy* was a significant positive independent predictor of the Dark Triad (β=.10, p < .05).

In a regression, *survivor guilt* was a strong positive independent predictor of the Light Triad (β=.47, p < .001), whereas *self-hate guilt* was a strong negative predictor of the Light Triad (β=-.59, p < .001). The Dark Triad showed the exact opposite pattern: *survival guilt* was a negative independent predictor of the Dark Triad (β=.35, p < .001), whereas *self-hate guilt* was a strong positive independent predictor of the Dark Triad (β=.35, p < .001).

In a regression, *perspective taking* (β=.48, p < .001), *inclusive identity* (β=.19, p < .001), and *personal growth* (β=.15, p < .001) all independently predicted the Light Triad. Note that *perspective taking* was especially predictive of the Light Triad.

In a regression, *perspective taking* was a strong negative independent predictor of the Dark Triad (β=.43, p < .001). None of the other facets of the Quiet Ego made an independent prediction on the Dark Triad.

**Selfishness, Aggression, and Moral Judgment**

In a regression, whereas *proactive aggression* independently negatively predicted the Light Triad (β=-.40, p < .001), *proactive aggression* independently positively predicted the Dark Triad (β=.53, p < .001). *Reactive aggression* was not independently related to either the Light or Dark Triad.

In a regression, both *egocentric selfishness* (β=-.39, p < .001) and *pathological selfishness* (β=-.28, p < .001) negatively independently predicted the Light Triad. Note that *adaptive selfishness* did not make an independent prediction on the Light Triad. In contrast, *pathological selfishness* (β=.56, p < .001), *egocentric selfishness* (β=.11, p < .001), and *adaptive selfishness* (β=.09, p < .001) all made independent positive predictions on the Dark Triad. However, note that the independent prediction of Pathological Selfishness on the Dark Triad was substantially greater than the other forms of selfishness.

**Religion, Spirituality, & Self-Transcendence**

In a regression, biosocial (β=.26, p < .001) and nature (β=.25, p < .001) routes to Death Transcendence independently predicted the Light Triad, whereas *religious* (β=.24, p < .01) and *creative* (β=.23, p < .01) routes to Death Transcendence independently predicted the Dark Triad.
